# Dynamics of Vector-Host Interactions in Avian Communities in Four Eastern Equine Encephalitis Virus Foci in the Northeastern U.S.

**DOI:** 10.1371/journal.pntd.0004347

**Published:** 2016-01-11

**Authors:** Goudarz Molaei, Michael C. Thomas, Tim Muller, Jan Medlock, John J. Shepard, Philip M. Armstrong, Theodore G. Andreadis

**Affiliations:** 1 Center for Vector Biology and Zoonotic Diseases, The Connecticut Agricultural Experiment Station, New Haven, Connecticut, United States of America; 2 Department of Mathematics, College of Science, Oregon State University, Corvallis, Oregon, United States of America; 3 Department of Biomedical sciences, College of Veterinary Medicine, Oregon State University, Corvallis, Oregon, United States of America; North Carolina State University, UNITED STATES

## Abstract

**Background:**

Eastern equine encephalitis (EEE) virus (*Togaviridae*, *Alphavirus*) is a highly pathogenic mosquito-borne zoonosis that is responsible for occasional outbreaks of severe disease in humans and equines, resulting in high mortality and neurological impairment in most survivors. In the past, human disease outbreaks in the northeastern U.S. have occurred intermittently with no apparent pattern; however, during the last decade we have witnessed recurring annual emergence where EEE virus activity had been historically rare, and expansion into northern New England where the virus had been previously unknown. In the northeastern U.S., EEE virus is maintained in an enzootic cycle involving the ornithophagic mosquito, *Culiseta melanura*, and wild passerine (perching) birds in freshwater hardwood swamps. However, the identity of key avian species that serve as principal virus reservoir and amplification hosts has not been established. The efficiency with which pathogen transmission occurs within an avian community is largely determined by the relative reservoir competence of each species and by ecological factors that influence contact rates between these avian hosts and mosquito vectors.

**Methodology and principle findings:**

Contacts between vector mosquitoes and potential avian hosts may be directly quantified by analyzing the blood meal contents of field-collected specimens. We used PCR-based molecular methods and direct sequencing of the mitochondrial *cytochrome b* gene for profiling of blood meals in *Cs*. *melanura*, in an effort to quantify its feeding behavior on specific vertebrate hosts, and to infer epidemiologic implications in four historic EEE virus foci in the northeastern U.S. Avian point count surveys were conducted to determine spatiotemporal host community composition. Of 1,127 blood meals successfully identified to species level, >99% of blood meals were from 65 avian hosts in 27 families and 11 orders, and only seven were from mammalian hosts representing three species. We developed an empirically informed mathematical model for EEE virus transmission using *Cs*. *melanura* abundance and preferred and non-preferred avian hosts. To our knowledge this is the first mathematical model for EEE virus, a pathogen with many potential hosts, in the northeastern U.S. We measured strong feeding preferences for a number of avian species based on the proportion of mosquito blood meals identified from these bird species in relation to their observed frequencies. These included: American Robin, Tufted Titmouse, Common Grackle, Wood Thrush, Chipping Sparrow, Black-capped Chickadee, Northern Cardinal, and Warbling Vireo. We found that these bird species, most notably Wood Thrush, play a dominant role in supporting EEE virus amplification. It is also noteworthy that the competence of some of the aforementioned avian species for EEE virus has not been established. Our findings indicate that heterogeneity induced by mosquito host preference, is a key mediator of the epizootic transmission of vector-borne pathogens.

**Conclusion and significance:**

Detailed knowledge of the vector-host interactions of mosquito populations in nature is essential for evaluating their vectorial capacity and for assessing the role of individual vertebrates as reservoir hosts involved in the maintenance and amplification of zoonotic agents of human diseases. Our study clarifies the host associations of *Cs*. *melanura* in four EEE virus foci in the northeastern U.S., identifies vector host preferences as the most important transmission parameter, and quantifies the contribution of preference-induced contact heterogeneity to enzootic transmission. Our study identifies Wood Thrush, American Robin and a few avian species that may serve as superspreaders of EEE virus. Our study elucidates spatiotemporal host species utilization by *Cs*. *melanura* in relation to avian host community. This research provides a basis to better understand the involvement of *Cs*. *melanura* and avian hosts in the transmission and ecology of EEE virus and the risk of human infection in virus foci.

## Author Summary

Eastern equine encephalitis (EEE) is a highly pathogenic mosquito-borne virus responsible for outbreaks of severe disease in humans and equines, causing high mortality and neurological impairment in most survivors. In the past, human disease outbreaks in the northeastern U.S. occurred sporadically with no apparent pattern; however, during the last decade, this region has experienced changes in the frequency of EEE virus activity with expansion into new localities. We studied vector-host interaction using molecular methods in order to: 1) quantify vector host-feeding preferences of *Culiseta melanura*, 2) identify key bird species that serve as frequent hosts for *Cs*. *melanura* and reservoir hosts for the virus, and 3) determine the extent to which these preferences shape virus transmission dynamics in four historic foci of EEE virus activity in Connecticut. We also examined avian population density and dynamics to determine spatiotemporal host community composition experienced by *Cs*. *melanura* and EEE virus. We developed an empirically informed mathematical model to describe enzootic transmission of EEE virus in a community of multiple avian hosts and the primary mosquito vector. To our knowledge this is the first mathematical model for EEE virus, a pathogen with many potential hosts, in the northeastern U.S. Our study clarifies the host associations of *Cs*. *melanura* in four foci of virus activity in the northeastern U.S., identifies vector host preferences as the most important transmission parameter, and quantifies the contribution of preference-induced contact heterogeneity to enzootic transmission. Our study also elucidates spatiotemporal host species utilization by *Cs*. *melanura* in relation to avian host abundance. We identified eight bird species (Wood Thrush, American Robin, Tufted Titmouse, Common Grackle, Chipping Sparrow, Black-capped Chickadee, Northern Cardinal, and Warbling Vireo) with a larger feeding index than the remaining birds, indicating these species were fed upon by *Cs*. *melanura* more frequently, and therefore are important in maintenance of EEE virus at these four foci. This research provides a basis for better understanding the involvement of *Cs*. *melanura* in the transmission and ecology of EEE virus, and the risk of human infection in virus foci.

## Introduction

Eastern equine encephalitis (EEE) virus (*Togaviridae*, *Alphavirus*) is responsible for outbreaks of severe disease in humans and equines, causing high mortality and neurological sequelae in most survivors [1,2]. EEE virus is maintained in an enzootic transmission cycle involving ornithophagic mosquitoes, specifically *Culiseta melanura* (Coquillett) (Diptera: Culicidae), and passerine birds in freshwater swamp foci [1–6]. In the past, disease outbreaks have occurred intermittently with no apparent pattern. Since 2003, however, the northeastern U.S. and southeastern Canada have experienced a resurgence of EEE virus activity with expansion into new regions [7]. These outbreaks occur when ecological factors and environmental conditions favor virus amplification followed by overflow into human and equine populations.

It is widely acknowledged that *Cs*. *melanura* feeds predominately on birds; however, the identity of key bird species that may serve as superspreaders of EEE virus has not been established in various virus foci [8–11]. Regional differences exist in the proportion of blood meals by *Cs*. *melanura* from various avian species that may be due to the availability and abundance of these birds among other ecological and physiological factors. Vector-host interaction studies conducted in EEE virus foci in the northeastern U.S. have identified >50 bird species as hosts for *Cs*. *melanura*, among which Wood Thrush and American Robin were most common [12–14]. Serological surveys also indicate that many of these bird species were frequently exposed to EEE virus [2,15]. The percentage of viral antibody was the highest for Wood Thrush, followed by American Robin, Ovenbird, and Swamp Sparrow in studies conducted in New Jersey and Massachusetts [2,16].

Successful transmission of EEE virus in avian host communities is governed by the abilities of host species to maintain, amplify, and transmit the virus to mosquito vectors (mainly *Cs*. *melanura*), and by ecological factors that influence contact rates between competent avian hosts and the mosquito vector. Earlier studies have identified a number of avian species as hosts for *Cs*. *melanura*. However, the potential for vector host preference to affect transmission of EEE virus in multiple foci has not been fully explored [12–14]. In this study, we investigated vector-host contact rates between *Cs*. *melanura* and avian hosts by identifying host species from blood meals, and the potential for heterogeneity in vector host preference to influence EEE virus transmission dynamics. The main objectives of this study were to 1) quantify vector host-feeding preferences in EEE virus foci, 2) identify key bird species that serve as frequent hosts for *Cs*. *melanura*, and as reservoir hosts for the virus, and 3) determine the extent to which these preferences shape the virus transmission dynamics. To achieve these objectives, we used PCR-based molecular methods and direct sequencing of the mitochondrial *cytochrome b* gene for profiling of blood meals in *Cs*. *melanura* to quantify its contact with vertebrate hosts, and to infer epidemiologic implications of its feeding behavior in four historic EEE virus foci in the northeastern U.S. We conducted avian point count surveys to determine spatiotemporal host community composition experienced by *Cs*. *melanura* and EEE virus. Finally, we developed a novel empirically informed mathematical model to describe enzootic transmission of EEE virus in a community of multiple avian hosts and the mosquito vector, *Cs*. *melanura*.

## Methods

### Study sites

**Fig 1:**
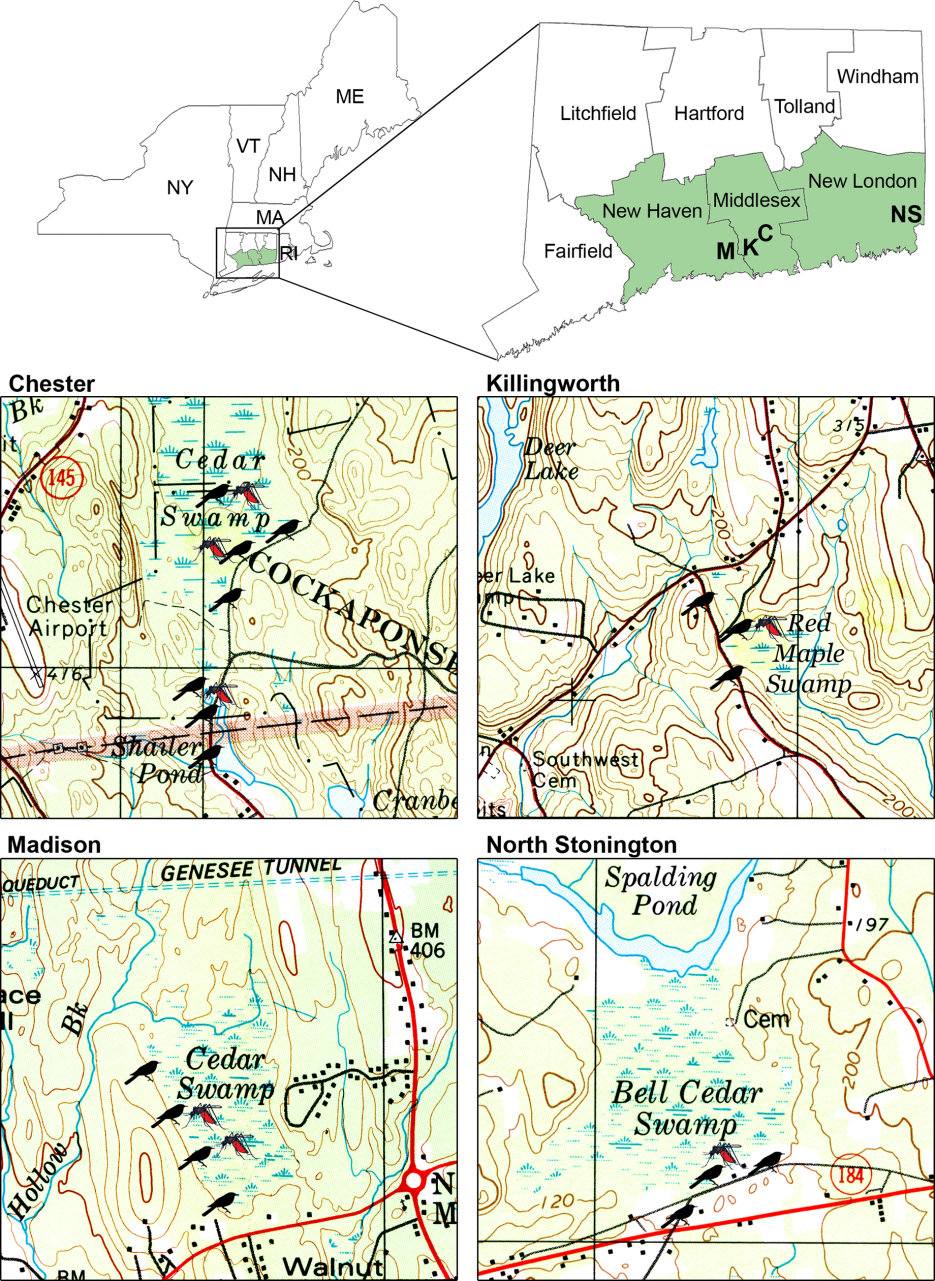
Location of mosquito collection sites and avian point count surveys in four historic EEE virus foci, Chester (C), Killingworth (K), Madison (M), and North Stonington (NS), CT, 2010–2011. Avian point count and mosquito trap locations are depicted by bird and mosquito images, respectively.

Field studies were conducted in four historic EEE virus foci, Chester, Killingworth, Madison, and North Stonington, CT (Fig 1). These four locations were considered to be virus foci because from 1996 to 2014, EEE virus was detected in 115 pools of mosquitoes, including 74 (63%) pools of *Cs*. *melanura*, the enzootic vector for EEE virus. North Stonington had the greatest number of positive pools (n = 47, 40.9%), followed by Chester (n = 42, 36.5%), Killingworth (n = 13, 11.3%), and Madison (n = 13, 11.3%). Majority of the positive pools were identified during 2009 (n = 56, 48.7%), which contributed to the rationales for initiating the present study during 2010–2011 (Table A in S1 Text). In 2010, four mosquito pools tested positive in mosquitoes from North Stonington but none from the other three sites. Interestingly, positive mosquito pools were identified in North Stonington in 8 years of the nearly two decades during which active mosquito surveillance has been conducted for EEE and other arboviruses in Connecticut.

#### Chester

The study site (41° 23.233' N; 72° 29.564' W) was located in the Cockaponset State Forest in the vicinity of a large seasonally flooded Atlantic white cedar swamp with a history of high *Cs*. *melanura* populations in Chester, CT. The swamp is dominated by Atlantic white cedar (*Chamaecyparis thyoides*), red maple (*Acer rubrum*), and yellow birch (*Betula alleghaniensis*) with a well-developed understory of mountain laurel (*Kalmia latifolia*). Sphagnum (*Sphagnum* spp.) and delicate fern moss (*Thudidium delicatulum*) are the principal ground cover, forming dense carpets throughout the swamp. Windthrow mounds with crypts supporting *Cs*. *melanura* larvae were commonly encountered. The study area surrounding the swamp consists of a variety of habitat types, including ponds, red maple swamps, and mixed evergreen-deciduous woodlands.

#### Killingworth

The study site (41° 20.217' N; 72° 34.322' W) was adjacent to a small seasonally flooded red maple swamp with scattered Atlantic white cedar in a residential neighborhood in Killingworth, CT. Tree cover is generally open with red maple as the dominant tree. Representative herbaceous and shrubby plants are sensitive fern (*Onoclea sensibilis*), skunk cabbage (*Symplocarpus foetidus*), spicebush (*Lindera benzoin*), and alders (*Alnus* spp.).

#### Madison

The study site (41° 21.628' N; 72° 39.131' W) was adjacent to a closed-canopy Atlantic white cedar swamp surrounded by a hardwood swamp and upland deciduous forest in Madison, CT. The dominant tree species include red maple, yellow birch, oaks (*Quercus* spp.), American beech (*Fagus grandifolia*), and tuliptree (*Liriodendron tulipifera*). The shrub layer consists of mountain laurel (*Kalmia latifolia*), witch hazel (*Hamamelis virginiana*), spicebush (*Lindera benzion*), sweet pepperbush (*Clethra alnifolia*), and highbush blueberry (*Vaccinium corymbosum*).

#### North Stonington

The study site (41° 26.175' N; 71° 49.845' W) was next to a large seasonally flooded Atlantic white cedar swamp, known as “Bell Cedar Swamp”, bordered by a mature stand of white pine (*Pinus strobus*), woodlands, and farmland in North Stonington, CT. The swamp is dominated by Atlantic white cedar, with red maple, yellow birch, and black gum *(Nyssa sylvatica*) scattered throughout the wetland forest. The understory is well developed and consists of spicebush, sweet pepperbush, highbush blueberry, swamp azalea (*Rhododendron viscosum*), and a groundcover of sphagnum mosses and ferns.

### Mosquitoes

A total of 6,234 female *Cs*. *melanura* were collected from the four EEE virus foci using 120 resting boxes (or 11”x11” stackable fiber nursery pots) placed on dry forested uplands within sight of red maple/Atlantic white cedar swamp habitats, and along the edges of these swamps in 8 sites, Chester 3, Killingworth 1, Madison 3, and North Stonington 1, during May through October, 2010–2011, and according to the established protocol [17] (Table 1). Greater numbers of *Cs*. *melanura* were collected during 2011 (total n = 4390; Chester 1936, Killingworth 777, Madison 1129, and North Stonington 548) than in 2010 (total n = 1844; Chester 548, Killingworth 545, Madison 577, and North Stonington 174). Multiple collection peaks were observed during the trapping season, which suggested 2–3 generations of *Cs*. *melanura* each year (Fig A in S1 Text). Resting boxes were examined daily, and a battery-powered handheld aspirator was used to collect engorged mosquitoes. Specimens were transported in coolers containing dry ice to the laboratory. Mosquitoes were then identified to species using a dissecting microscope and an identification key [18]. Specimens with visible blood meals were transferred to 1.5 mL microtubes, labeled with a unique number, and stored in an ultra-low temperature freezer.

**Table 1:** Site coordinates; number of resting box sites, resting boxes, engorged *Cs*. *melanura*, avian point count sites, avian point counts, and avian species encountered at EEE virus foci in Chester, Killingworth, Madison, and North Stonington, 2010–2011.

Study Site	Lat/Long Coordinates	No. of Resting Box Sites	No. of Resting Boxes	No. of Engorged *CS*. *melanura*	No. of Avian Point Count Sites	No. of Avian Point Counts	No. of Avian Species Encountered
Chester	41° 23.233' N 72° 29.564' W	3	45	348	7	301	99
Killingworth	41° 20.217' N 72° 34.322' W	1	15	249	3	132	66
Madison	41° 21.628' N 72° 39.131' W	3	45	361	4	172	66
North Stonington	41° 26.175' N 71° 49.845' W	1	15	169	3	84	68
Total		8	120	1127	17	689	

### DNA extraction and amplification

Mosquito abdomens were removed with the aid of a dissecting microscope and disposable razor blades for blood meal analysis. DNA was extracted from the abdominal content of engorged mosquitoes individually by using DNAzol BD (Molecular Research Center, Cincinnati, OH, USA) according to the manufacturer’s recommendation with some modifications as described elsewhere [13,19]. Extracted DNA from the mosquito blood meals served as DNA templates in subsequent polymerase chain reaction assays with primers based on vertebrate mitochondrial *cytochrome b* sequences according to published protocols [13,19]. Sequencing of both DNA strands was carried out on 3730xL DNA Analyzers, using Big Dye chemistries (Applied Biosystems Inc., Grand Island, NY) at the Keck Sequencing Facility, Yale University, New Haven, CT. Sequences were analyzed and annotated using ChromasPro version 1.7.5 (Technelysium Pty Ltd., Tewantin, Australia), and identified by comparison to the GenBank DNA sequence database utilizing the BLAST search (BLASTN) of the National Center for Biotechnology Information [20].

### Avian point count survey

Avian point count surveys were conducted in the study sites weekly from April through October, 2010–2011 in order to assess species composition and relative spatial and temporal abundance of bird species, according to the previously described protocols [21,22] (Table 1). A skilled observer knowledgeable of the identification and vocalizations (i.e. songs and call notes) of the local bird fauna conducted the point count surveys. Three 100 m-diameter point count circles, located 50–200 m apart, were established at each resting box site with the aid of a Garmin eTerx 10 GPS unit (Garmin International, Inc., Olathe, KS) and a rangefinder (Bushnell Outdoor Products, Overland Park, KS). To estimate bird distances within the point count circles, flagging tape was affixed to trees or shrubs at distances of 15, 30, and 50 m. Landmarks within the point count circles were also used to estimate distances. Counts began shortly after sunrise under favorable weather conditions, when bird activity and vocalization are highest. The observer approached the point count with as little disturbance as possible and began counting birds upon arrival at the site. With the aid of binoculars and a stopwatch, the observer recorded the distance and number of bird species seen and heard during a 15-minute period. Status and habitat codes, including observations of nestlings, fledglings, and juvenile birds, and individuals detected outside or flying over the point count circles, were recorded. Temperature and wind speed was measured with a hand-held anemometer (La Crosse Technology, La Crosse, WI) and recorded on the form along with the start and finish times (Fig B in S1 Text). Bird nomenclature followed the 7th edition of the “American Ornithologists’ Union” Checklist [23].

### Modeling methods

The modeling methods are briefly described here, and more detailed information is provided in the “Supporting Information”. The data for each of the four EEE virus foci was pooled to increase sample size and to illustrate general trends. Across the four locations, eight bird species were selected based on their high abundance in bird counts or high prevalence as the source of blood meals, including Wood Thrush (*Hylocichla mustelina*), American Robin (*Turdus migratorius*), Tufted Titmouse (*Baeolophus bicolor*), Common Grackle (*Quiscalus quiscula*), Chipping Sparrow (*Spizella passerina*), Black-capped Chickadee (*Poecile atricapillus*), Northern Cardinal (*Cardinalis cardinalis*), and Warbling Vireo (*Vireo gilvus*). The remaining bird species at each location were combined into a ninth category of other birds. Our model simulates the dynamics of EEE virus in each of these eight bird species, the remaining birds and *Cs*. *melanura*, over a season of 180 days. We chose this time interval to simulate a typical mosquito activity season.

The feeding index was used to model the preference of *Cs*. *melanura* for the different bird species [24,25]. The feeding index assesses the proportion of blood meals from a host species relative to the abundance of that species in the host community, or the relative likelihood of a blood meal on a given bird species per bird of that species. Thus a feeding index shows the relative preference for one bird species compared to the other species examined. As a result, we chose the ninth category, other birds, as the frame of reference for the other species, assigning its feeding index the value of 1. We calculated the feeding index for the eight bird species from the bird count and blood meal data collected in this study.

The force of infection is defined as the per-capita rate, or hazard, at which susceptible hosts become infected, with a separate force of infection for each bird species and one for the mosquitoes. The number of female mosquitoes, the feeding index and bird abundances determine the relative number of mosquito bites on each bird species. The proportion of infectious mosquitoes and the vector-to-host transmission rate provide the force of infection for the bird species, while the proportions of each infectious bird and the host-to-vector transmission rate give the mosquito force of infection. We assumed that host-to-vector and vector-to-host transmission rates were constant across all bird species, due to a lack of comprehensive information on species-specific transmission rates. Statistical sampling models were used to account for sampling error due to small numbers of bird counts and blood meals for some species. Utilizing Markov chain Monte Carlo methods, 1000 samples were selected for both the counts and blood meals. For each of these samples, the feeding index of the selected bird species were calculated, and the median and inner 95% quantiles were reported.

For each of the selected bird species, we used a standard SIR sub-model to simulate the EEE virus transmission dynamics, with the population of each species divided into susceptible, infectious, and recovered compartments. We assumed that vectors do not recover from infection, using a SI sub-model. Each sub-model includes births and deaths, with birth balancing deaths leaving population sizes constant. The number of susceptible hosts increases according to their birth rates, and decreases due to infection and death. For simplicity, we assumed that there was no death due to infection in birds and mosquitoes, so that death is only due to background mortality, and that the population size of each bird species and mosquitoes remained constant throughout the simulation period. Mosquitoes and each bird species transition from susceptible to infectious according to their forces of infection; birds transition from infectious to recovered at a recovery rate of 1 per day, i.e. 1 day mean duration of infectious viremia. The model then simulated for a period of 180 days, starting with all bird species completely susceptible and one tenth of one percent of mosquitoes infectious.

Studies suggest that vector-to-host transmission is guaranteed when an infectious vector feeds from a host [26], and thus the vector-to-host transmission rate was set to 1. Limited data exists regarding the value of the host-to-vector transmission rate among the various host species. In order to focus the analysis on the effect of variable biting rates on the model's output, the host-to-vector transmission rate is assumed to be constant across all of the host species. The all-species host-to-vector transmission rate was determined by fitting the model proportion of birds that became infected over the model period to the observed proportion of seropositives amongst the various bird species from a previous study [15]. For bird species that were not present in the previous study, the mean proportion of seropositives from that study was used.

## Results

### Vertebrate host choice by *Culiseta melanura*

A total of 1,798 *Cs*. *melanura* with visible blood meals were collected from the four virus foci, and blood meal sources were identified in 1,127 (62.7%) specimens by DNA sequencing. These included Chester 348 of 581 (59.9%), Killingworth 249 of 364 (68.4%), Madison 361 of 598 (60.4%), and North Stonington 169 of 255 (66.3%). The remaining blood-fed *Cs*. *melanura* either did not produce visible amplification products or the sequencing results were insufficiently conclusive to assign a host species. In addition to *Cs*. *melanura*, 372 engorged specimens of 12 species in the genera of *Aedes*, *Anopheles*, *Coquillettidia*, *Culex*, *Culiseta*, and *Ochlerotatus* were collected, and blood meal analyses conducted (Table B in S1 Text). However, because the focus of the present study was on *Cs*. *melanura*, the principal mosquito vector of EEE virus, results of these analyses are not presented here.

Of the 1,127 engorged *Cs*. *melanura* blood meals that were successfully linked to a vertebrate host at the species level, 99.4% were from avian hosts, comprising 65 species from 27 families and 11 orders. Passeriformes constituted the most numerous hosts representing 97.5% of avian blood meals. Comparatively few Cuculiformes (1.0%), Columbiformes (0.6%), Accipitriformes (0.3%), Strigiformes (0.2%), and six other avian orders were additionally identified. Among taxonomic families, Turdidae (thrushes) served as the most frequent hosts (33.4%), followed by Paridae (chickadees and titmice, 18.6%), Cardinalidae (cardinals and tanagers, 10.9%), Icteridae (blackbirds, 9.5%), Vireonidae (vireos, 8.0%), and 22 other avian families (Table 2). Four mammalian species belonging to the Cervidae (White-tailed Deer), Bovidae (Domestic Cow and Sheep) and Sciuridae (Eastern Gray Squirrel) were also identified in individual or mixed blood meals.

**Table 2:** Number and percentage of avian families (n = 27) as host choice for *Cs*. *melanura* in Chester, Killingworth, Madison, and North Stonington, CT, May through October, 2010–2011.

Order/Family	Chester	Killingworth	Madison	North Stonington	Total	%
**Passeriformes**						
Turdidae (Thrushes)	55	116	159	44	374	33.4
Paridae (Chickadees and Titmice)	99	55	39	15	208	18.6
Cardinalidae (Cardinals and Tanagers)	23	23	42	34	122	10.9
Icteridae (Blackbirds)	40	14	41	11	106	9.5
Vireonidae (Vireos)	58	3	18	10	89	8.0
Emberizidae (New World Sparrow)	16	13	19	25	73	6.5
Parulidae (Wood-Warblers)	16	8	18	10	52	4.6
Mimidae (Mockingbirds and Thrashers)	6	4	7	4	21	1.9
Fringillidae (Finches and Allies)	5	1	2		8	0.7
Polioptilidae (Gnatcatchers)	6	2			8	0.7
Sturnidae (Starlings)	3		5		8	0.7
Troglodytidae (Wrens)	1	1	3	3	8	0.7
Tyrannidae (Tyrant Flycatchers)	2	1	1		4	0.4
Hirundinidae (Swallows)	1		1	1	3	0.3
Passeridae (Old World Sparrow)		3			3	0.3
Bombycillidae (Waxwings)	1		1		2	0.2
Corvidae (Jays and Crows)	1				1	0.1
**Cuculiformes**						
Cuculidae (Cuckoos)	2	1	1	7	11	1.0
**Columbiformes**						
Columbidae (Pigeons and Doves)	5	1		1	7	0.6
**Accipitriformes**						
Accipitridae (Hawks and Eagles)			2	2	4	0.3
**Strigiformes**						
Strigidae (Typical Owls)	2				2	0.2
**Anseriformes**						
Anatidae (Ducks, Geese, and Swans)	1				1	0.1
**Pelecaniformes**						
Ardeidae (Herons, Bitterns, and Allies)		1			1	0.1
**Galliformes**						
Phasianidae (Turkeys, Grouse, and Quail)			1		1	0.1
**Piciformes**						
Picidae (Woodpeckers)		1			1	0.1
**Gruiformes**						
Rallidae (Rails, Gallinules, and Coots)				1	1	0.1
**Charadriiformes**						
Scolopacidae (Sandpipers and Allies)	1				1	0.1
Total	344	248	360	168	1120	

#### Chester

Analysis of 348 engorged *Cs*. *melanura* for which the host sources were successfully identified to species level, revealed that 344 (98.9%), and four (1.1%) obtained blood meals from avian and mammalian hosts, respectively, including 40 avian and one mammalian species (White-tailed Deer). Tufted Titmouse was the most frequently identified source of blood comprising 22.4% (n = 78) of all vertebrate-derived blood meals, followed by American Robin (n = 38, 10.9%), Common Grackle (n = 28, 8.0%), Warbling Vireo (n = 23, 6.6%), and Red-eyed Vireo (n = 23, 6.6%) (Tables 3 and C in S1 Text). Four specimens were identified with mixed blood meals from avian and mammalian species. The identity of the avian hosts in mixed blood meals was determined as Common Grackle (n = 2), and Tufted Titmouse and Wood Thrush (each n = 1). White-tailed Deer was identified as the only mammalian host in all four specimens with mixed blood meals.

**Table 3:** Number and percentage of avian- and mammalian-derived blood meals identified from *Cs*. *melanura* in Chester, CT, May through October, 2010–2011. *R.C. = Residency codes: P, permanent resident (found year round in the state); S, summer resident (present in the state during the nesting season); T, transient.

			May	June	July	Aug	Sept	Oct	
Vertebrate Host	Scientific Name	R. C.*	No. (%)	No. (%)	No. (%)	No. (%)	No. (%)	No. (%)	Total
**Avian**									
Tufted Titmouse	*Baeolophus bicolor*	P	1 (100)	7 (6.5)	11(14.3)	29 (29.6)	26 (44.1)	4 (66.7)	78
American Robin	*Turdus migratorius*	P, T		15 (14.0)	10 (13.0)	10 (10.2)	3 (5.1)		38
Common Grackle	*Quiscalus quiscula*	P, T		22 (20.6)	1 (1.3)	4 (4.1)	1 (1.7)		28
Warbling Vireo	*Vireo gilvus*	S		7 (6.5)	16 (20.8)				23
Red-eyed Vireo	*Vireo olivaceus*	S		5 (4.7)		10 (10.2)	8 (13.6)		23
Black-capped Chickadee	*Poecile atricapillus*	P		6 (5.6)	7 (9.1)	8 (8.2)			21
Wood Thrush	*Hylocichla mustelina*	S		2 (1.9)	4 (5.2)	3 (3.1)	5 (8.5)	2 (33.3)	16
Chipping Sparrow	*Spizella passerina*	S		4 (3.7)	3 (3.9)	5 (5.1)	2 (3.4)		14
Yellow-throated Vireo	*Vireo flavifrons*	S		4 (3.7)		2 (2.0)	6 (10.2)		12
Northern Cardinal	*Cardinalis cardinalis*	P		4 (3.7)		4 (4.1)	1 (1.7)		9
Scarlet Tanager	*Piranga olivacea*	S		3 (2.8)	5 (6.5)		1 (1.7)		9
Ovenbird	*Seiurus aurocapilla*	S		4 (3.7)	3 (3.9)	1 (1.0)			8
Blue-gray Gnatcatcher	*Polioptila caerulea*	S		2 (1.9)	1 (1.3)	3 (3.1)			6
Gray Catbird	*Dumetella carolinensis*	S		4 (3.7)	2 (2.6)				6
American Goldfinch	*Spinus tristis*	P				5 (5.1)			5
Other Avian Species				14 (13.1)	14 (18.2)	14 (14.3)	6 (10.2)		48
**Mammalian**									
White-tailed Deer	*Odocoileus virginianus*	P		4 (3.7)					4
Total			1	107	77	98	59	6	348

#### Killingworth

Of the 249 engorged *Cs*. *melanura*, for which the host sources were successfully identified to species level, 248 (99.6%) and one (0.4%) acquired blood meals from avian and mammalian hosts, respectively, representing 35 avian and one mammalian species (White-tailed Deer). Wood Thrush was the most frequently identified source of blood comprising 26.9% (n = 67) of all vertebrate-derived blood meals, followed by American Robin (n = 46, 18.5%), Tufted Titmouse (n = 39, 15.7%), Black-capped Chickadee (n = 16, 6.4%), and Northern Cardinal (n = 12, 4.8%) (Tables 4 and D in S1 Text).

**Table 4:** Number and percentage of avian- and mammalian-derived blood meals identified from *Cs*. *melanura* in Killingworth, CT, May through October, 2010–2011. *R.C. = Residency codes: P, permanent resident (found year round in the state); S, summer resident (present in the state during the nesting season); T, transient.

			May	June	July	Aug	Sept	Oct	
Vertebrate Host	Scientific Name	R. C.*	No. (%)	No. (%)	No. (%)	No. (%)	No. (%)	No. (%)	Total
Wood Thrush	*Hylocichla mustelina*	S	1 (6.3)	3 (8.1)	6 (9.7)	45 (46.9)	12 (42.9)		67
American Robin	*Turdus migratorius*	P, T	6 (37.5)	15 (40.5)	13 (21.0)	8 (8.3)	2 (7.1)	2 (20.0)	46
Tufted Titmouse	*Baeolophus bicolor*	P	1 (6.3)	7 (18.9)	19 (30.6)	8 (8.3)	4 (14.3)		39
Black-capped Chickadee	*Poecile atricapillus*	P	1 (6.3)	4 (10.8)	7 (11.3)	3 (3.1)		1 (10.0)	16
Northern Cardinal	*Cardinalis cardinalis*	P		1 (2.7)	3 (4.8)	5 (5.2)	1 (3.6)	2 (20.0)	12
Common Grackle	*Quiscalus quiscula*	P, T		2 (5.4)	3 (4.8)	4 (4.2)	1 (3.6)	1 (10.0)	11
Scarlet Tanager	*Piranga olivacea*	S	3 (18.8)	1 (2.7)	1 (1.6)	5 (5.2)			10
Chipping Sparrow	*Spizella passerina*	S	1 (6.3)		2 (3.2)	4 (4.2)	1 (3.6)	1 (10.0)	9
Gray Catbird	*Dumetella carolinensis*	S			2 (3.2)	1 (1.0)	1 (3.6)		4
Other Avian Species			3 (18.8)	4 (10.8)	6 (9.7)	13 (13.5)	6 (21.4)	2 (20.0)	34
**Mammalian**									
White-tailed Deer	*Odocoileus virginianus*	P						1 (10.0)	1
Total			16	37	62	96	28	10	249

#### Madison

Analysis of 361 engorged *Cs*. *melanura* for which the host sources were successfully identified to species level, revealed that 360 (99.7%) and one (0.3%) obtained blood meals from avian and mammalian hosts, respectively, representing 42 avian and one mammalian species (Sheep). Wood Thrush was the most frequent source of blood meal comprising 28.5% (n = 103) of all vertebrate-derived blood meals, followed by American Robin (n = 51, 14.1%), Common Grackle (n = 31, 8.6%), Tufted Titmouse (n = 21, 5.8%), and Black-capped Chickadee (n = 18, 5.0%) (Tables 5 and E in S1 Text).

**Table 5:** Number and percentage of avian- and mammalian-derived blood meals identified from *Cs*. *melanura* in Madison, CT, May through October, 2010–2011. (*R.C. = Residency codes: P, permanent resident (found year round in the state); S, summer resident (present in the state during the nesting season); T, transient.

			May	June	July	Aug	Sept	Oct	
Vertebrate Host	Scientific Name	R. C.*	No. (%)	No. (%)	No. (%)	No. (%)	No. (%)	No. (%)	Total
Wood Thrush	*Hylocichla mustelina*	S		11 (11.7)	9 (13.8)	56 (40.3)	27 (52.9)		103
American Robin	*Turdus migratorius*	P, T	2 (28.6)	17 (18.1)	20 (30.8)	10 (7.2)	2 (3.9)		51
Common Grackle	*Quiscalus quiscula*	P, T		9 (9.6)	3 (4.6)	17 (12.2)	2 (3.9)		31
Tufted Titmouse	*Baeolophus bicolor*	P	1 (14.3)	6 (6.4)	4 (6.2)	9 (6.5)	1 (2.0)		21
Black-capped Chickadee	*Poecile atricapillus*	P		8 (8.5)	3 (4.6)	5 (3.6)	2 (3.9)		18
Northern Cardinal	*Cardinalis cardinalis*	P		7 (7.4)	3 (4.6)	3 (2.2)	3 (5.9)	1 (20.0)	17
Scarlet Tanager	*Piranga olivacea*	S	1 (14.3)	7 (7.4)		8 (5.8)	1 (2.0)		17
Chipping Sparrow	*Spizella passerina*	S		7 (7.4)	4 (6.2)	4 (2.9)		1 (20.0)	16
Red-eyed Vireo	*Vireo olivaceus*	S	1 (14.3)	3 (3.2)	5 (7.7)	4 (2.9)			13
Gray Catbird	*Dumetella carolinensis*	S		4 (4.3)	2 (3.1)			1 (20.0)	7
Rose-breasted Grosbeak	*Pheucticus ludovicianus*	S	1 (14.3)	1 (1.1)		3 (2.2)	1 (2.0)		6
European Starling	*Sturnus vulgaris*	P	1 (14.3)		1 (1.5)	1 (0.7)	1 (2.0)	1 (20.0)	5
Ovenbird	*Seiurus aurocapilla*	S		2 (2.1)		2 (1.4)	1 (2.0)		5
Other Avian Species				12 (12.8)	11 (16.9)	17 (12.2)	9 (17.6)	1 (20.0)	50
**Mammalian**									
Sheep	*Ovis aries*						1 (2.0)		1
Total	* *		7	94	65	139	51	5	361

#### North Stonington

Of the 169 engorged *Cs*. *melanura* for which the host sources were successfully identified to species level, 168 (99.4%) and one (0.6%) acquired blood meals from avian and mammalian hosts, respectively, representing 32 avian and one mammalian species (Eastern Gray Squirrel). Chipping Sparrow was the most frequent source of blood meal comprising 13.0% (n = 22) of all vertebrate-derived blood meals, followed by Northern Cardinal (n = 21, 12.4%), American Robin (n = 20, 11.8%), Wood Thrush (n = 20, 11.8%), and Tufted Titmouse (n = 10, 5.9%) (Tables 6 and F in S1 Text).

**Table 6:** Number and percentage of avian- and mammalian-derived blood meals identified from *Cs*. *melanura* in North Stonington, CT, May through October, 2010–2011. *R.C. = Residency codes: P, permanent resident (found year round in the state); S, summer resident (present in the state during the nesting season); T, transient.

			May	June	July	Aug	Sept	Oct	
Vertebrate Host	Scientific Name	R. C.*	No. (%)	No. (%)	No. (%)	No. (%)	No. (%)	No. (%)	Total
Chipping Sparrow	*Spizella passerina*	S		7 (22.6)	6 (13.0)	7 (12.7)	2 (6.9)		22
Northern Cardinal	*Cardinalis cardinalis*	P		1 (3.2)	4 (8.7)	11 (20.0)	5 (17.2)		21
American Robin	*Turdus migratorius*	P, T		5 (16.1)	5 (10.9)	7 (12.7)	3 (10.3)		20
Wood Thrush	*Hylocichla mustelina*	S			4 (8.7)	8 (14.5)	8 (27.6)		20
Tufted Titmouse	*Baeolophus bicolor*	P		4 (12.9)	5 (10.9)		1 (3.4)		10
Common Grackle	*Quiscalus quiscula*	P, T		2 (6.5)	1 (2.2)	4 (7.3)			7
Red-eyed Vireo	*Vireo olivaceus*	S		2 (6.5)	3 (6.5)	1 (1.8)			6
Yellow-billed Cuckoo	*Coccyzus americanus*	S	2 (40.0)		3 (6.5)	1 (1.8)			6
Black-capped Chickadee	*Poecile atricapillus*	P		1 (3.2)	2 (4.3)	1 (1.8)		1 (33.3)	5
Rose-breasted Grosbeak	*Pheucticus ludovicianus*	S			2 (4.3)	3 (5.5)			5
Scarlet Tanager	*Piranga olivacea*	S		2 (6.5)	2 (4.3)		1 (3.4)		5
Gray Catbird	*Dumetella carolinensis*	S	1 (20.0)	1 (3.2)		2 (3.6)			4
Pine Warbler	*Setophaga pinus*	S				3 (5.5)	1 (3.4)		4
Veery	*Catharus fuscescens*	S		1 (3.2)	2 (4.3)		1 (3.4)		4
Warbling Vireo	*Vireo gilvus*	S			3 (6.5)	1 (1.8)			4
House Wren	*Troglodytes aedon*	S			1 (2.2)	1 (1.8)	1 (3.4)		3
Other Avian Species			2 (40.0)	5 (16.1)	3 (6.5)	5 (9.1)	5 (17.2)	2 (66.7)	22
**Mammalian**									
Eastern Gray Squirrel	*Sciurus carolinensis*						1 (3.4)		1
Total	* *		5	31	46	55	29	3	169

### Avian population analysis

A total of 37 avian families in 14 orders were encountered at the study sites during the point count survey with differing frequencies calculated over 7 months, April to October. The most frequent avian order was Passeriformes (86.2% of all birds) with 20 families including Paridae (chickadees and titmice, 17.7%), Icteridae (blackbirds, 9.7%), Turdidae (thrushes, 8.8%), Parulidae (wood warblers, 8.1%), and Emberizidae (New World Sparrow, 6.7%). Other relatively frequent avian orders included Piciformes ([Picidae (woodpeckers)], 5.2%), Anseriformes ([Anatidae (ducks, geese, and swans)], 3.3%), and Columbiformes ([Columbidae (doves and pigeons)], 1.6%) (Table G in S1 Text).

#### Chester

A total of 99 avian species in 35 families were encountered during 43 avian point counts at 7 sites. Tufted Titmouse (Passeriformes, Paridae) was the most frequent avian species encountered (Average Frequency of 1.0), followed by Black-capped Chickadee (0.95) (Passeriformes, Paridae), American Goldfinch (0.88) (Passeriformes, Fringillidae), American Robin (0.88) (Passeriformes, Turdidae), Blue Jay (0.88) (Passeriformes, Corvidae) and then 94 other avian species. Of 99 avian species encountered, 37 species served as hosts for *Cs*. *melanura* (Table H in S1 Text).

#### Killingworth

A total of 66 avian species representing 28 families were encountered during 44 avian point counts at 3 sites. Tufted Titmouse was identified as the most frequent avian species (Average Frequency of 1.0), followed by Northern Cardinal (0.93) (Passeriformes, Cardinalidae), American Robin (0.86), Red-bellied Woodpecker (0.86) (Piciformes, Picidae), Black-capped Chickadee (0.84), and then 61 other bird species. Of the 66 avian species encountered, 27 species served as hosts for *Cs*. *melanura* (Table I in S1 Text).

#### Madison

A total of 66 avian species in 25 families were encountered during 43 avian point counts at 4 sites. We identified Tufted Titmouse with the highest frequency of encounter during the survey (Average Frequency of 0.93). Other frequently observed avian species included Black-capped Chickadee (0.79), White-breasted Nuthatch (0.72) (Passeriformes, Sittidae), Blue Jay (0.70), Downy Woodpecker (0.63) (Piciformes, Picidae), and 61 other avian species. Of the 66 avian species encountered, 31 served as hosts for *Cs*. *melanura* (Table J in S1 Text).

#### North Stonington

A total of 68 avian species representing 33 families were encountered during 28 avian point counts at 3 sites. Black-capped Chickadee was the most frequently observed species (Average Frequency of 1.0), followed by Northern Cardinal (0.89), American Robin (0.86), Tufted Titmouse (0.86), Gray Catbird (0.82) (Passeriformes, Mimidae), and then 63 other bird species. Of the 68 avian species encountered, 25 species served as hosts for *Cs*. *melanura* (Table K in S1 Text).

### *Culiseta melanura* blood feeding in relation to avian frequencies

We compared percentage of avian-derived blood meals for *Cs*. *melanura* with average avian frequencies in the four EEE virus foci.

#### Chester

**Fig 2:**
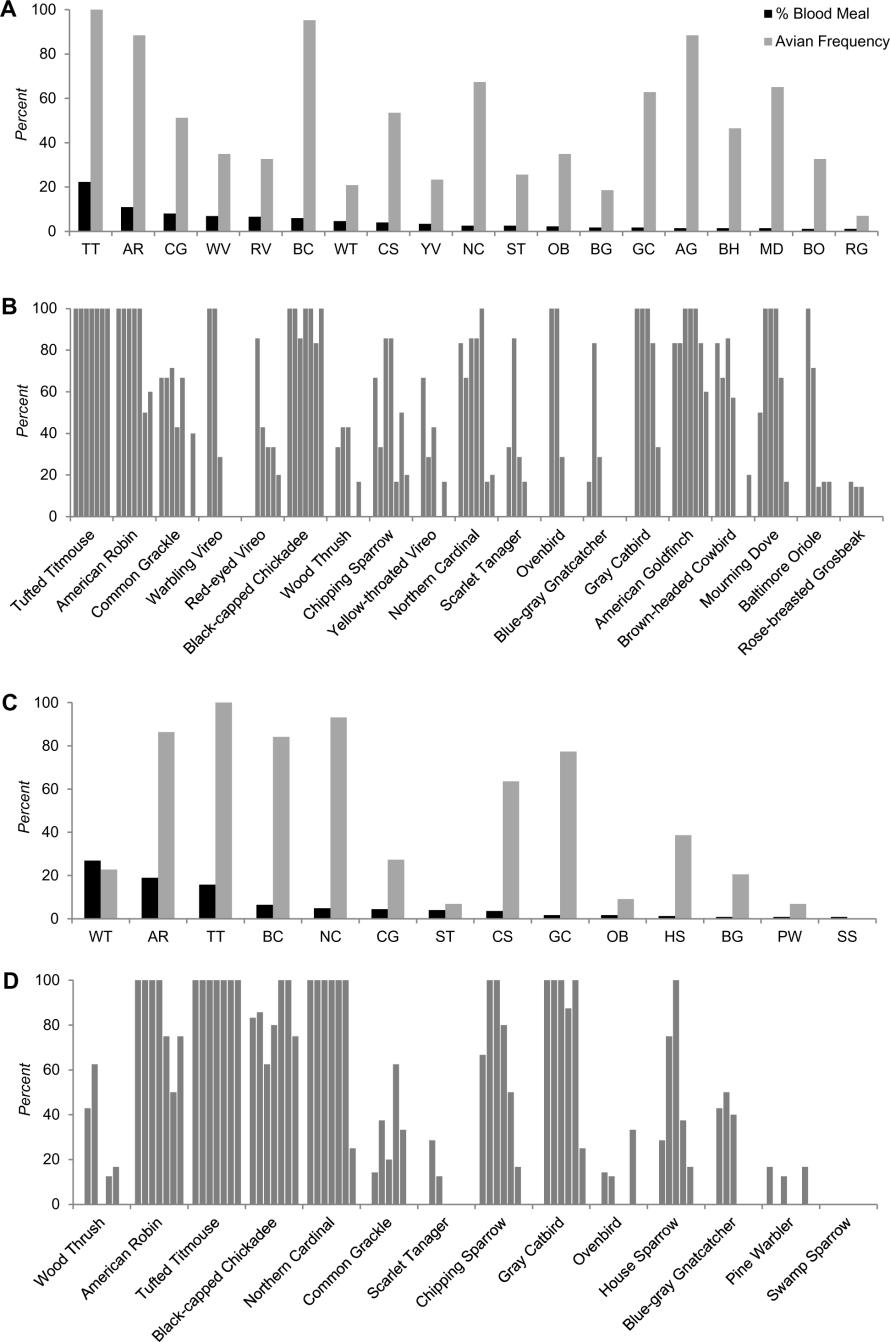
Percentage of avian-derived blood meals for *Cs*. *melanura* compared with average avian frequencies in Chester, CT (A), and Killingworth, CT (C), May through October, 2010–2011. Monthly frequencies of avian species based on point count data in Chester, CT (B), and Killingworth, CT (D), April through October, 2010–2011.

Frequencies of observation for bird species were recorded during the two- year avian point count surveys (Table H in S1 Text). The percentage of *Cs*. *melanura* that acquired blood meals from avian species such as Tufted Titmouse, American Robin, Black-capped Chickadee, Northern Cardinal, Common Grackle, and a few other birds were as expected based on their frequencies, however, for other avian species such as Eastern Towhee, Black-and-white Warbler, Eastern Wood-Pewee, Barn Swallow, and Chipping Sparrow, it was considerably lower (Fig 2A and 2B; Tables 3 and C in S1 Text). We did not identify any blood meal derived from Blue Jay, White-breasted Nuthatch, Downy Woodpecker, Red-bellied Woodpecker, Eastern Phoebe, and several others, despite their relatively higher frequencies of observation in avian point count survey.

#### Killingworth

We identified several avian species with greater frequencies of observation during point count survey (Table I in S1 Text). The percentage of *Cs*. *melanura* blood meals from bird species such as American Robin, Tufted Titmouse, Black-capped Chickadee, Northern Cardinal, and several other bird species was as expected based on their frequencies of observation. Other avian species such as Red-bellied Woodpecker, American Goldfinch, Brown-headed Cowbird, Mourning Dove, and a number of other avian species were underrepresented in blood meals (Fig 2C and 2D; Tables 4 and D in S1 Text). Downy Woodpecker, Blue Jay, White-breasted Nuthatch, Carolina Wren, and a number of other birds were identified with a relatively higher frequency of observations, but no blood meals from these species were identified.

#### Madison

**Fig 3:**
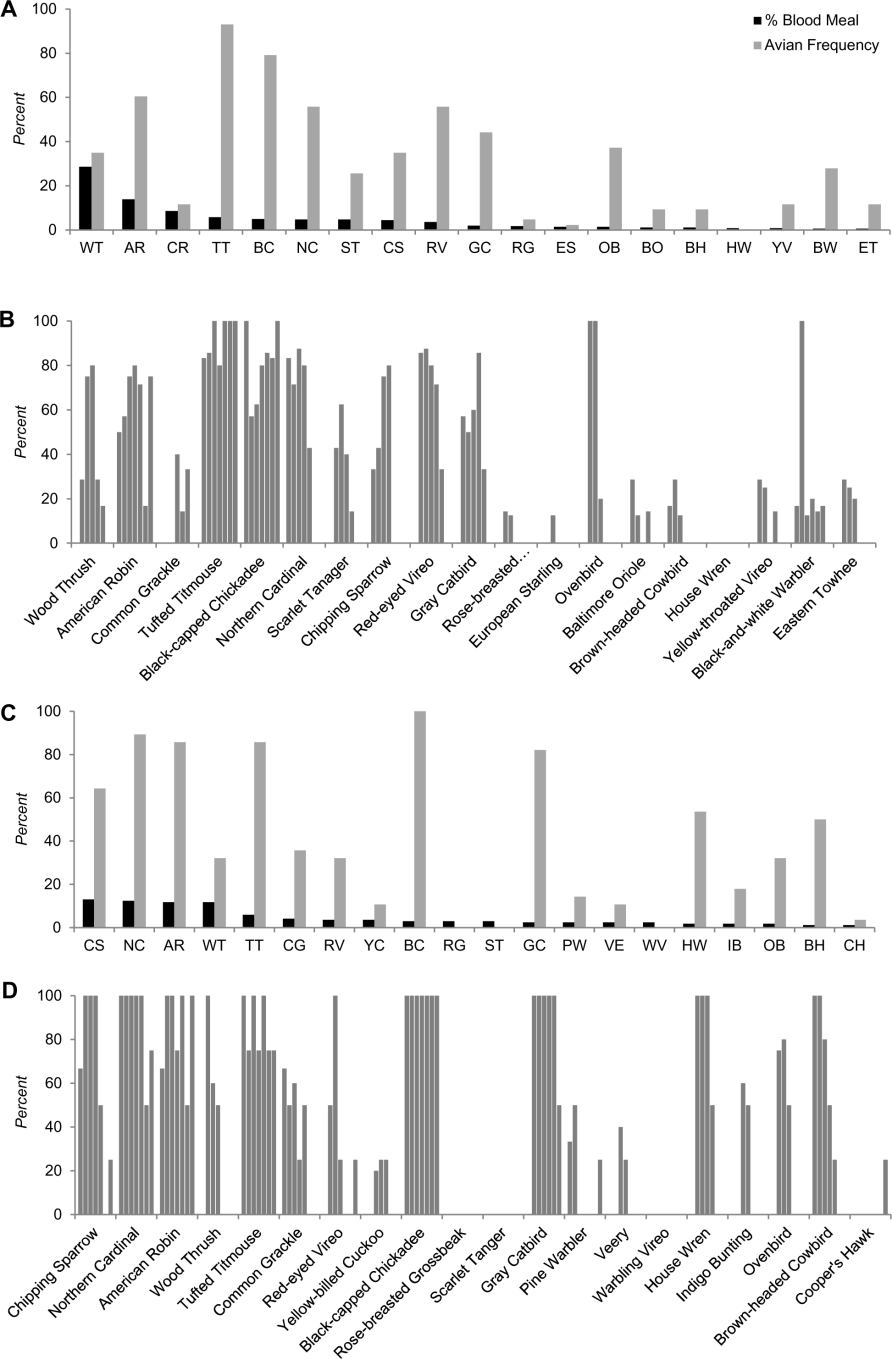
Percentage of avian-derived blood meals for *Cs*. *melanura* compared with average avian frequencies in Madison, CT (A), and North Stonington, CT (C), May through October, 2010–2011. Monthly frequencies of avian species based on point count data in Madison, CT (B), and North Stonington, CT (D), April through October, 2010–2011.

Several bird species were commonly observed during the avian point count surveys (Table J in S1 Text). The percentage of *Cs*. *melanura* blood meals from American Robin, Tufted Titmouse, Black-capped Chickadee, Northern Cardinal, Red-eyed Vireo, Gray Catbird, and a few other birds was as expected based on their frequency of observation (Fig 3A and 3B; Tables 5 and E in S1 Text). However, we did not identify blood meals from White-breasted Nuthatch, Blue Jay, Downy Woodpecker, Red-bellied Woodpecker, American Goldfinch, and several other bird species despite their observed frequencies.

#### North Stonington

Several avian species were frequently observed during the avian point count survey (Table K in S1 Text), and the proportion of *Cs*. *melanura* blood meals from some of these bird species was as expected based on their frequency of observation (Fig 3C and 3D; Tables 6 and F in S1 Text). Although Chipping Sparrow was identified as the most frequent source of blood meal at this site, this avian species had the 8^th^ highest frequency of observation (Table K in S1 Text). American Goldfinch, House Sparrow, Blue Jay, Downy Woodpecker, and Fish Crow were also observed with relatively higher frequencies, but we did not identify blood meals originated from these birds.

### Modeling

**Fig 4:**
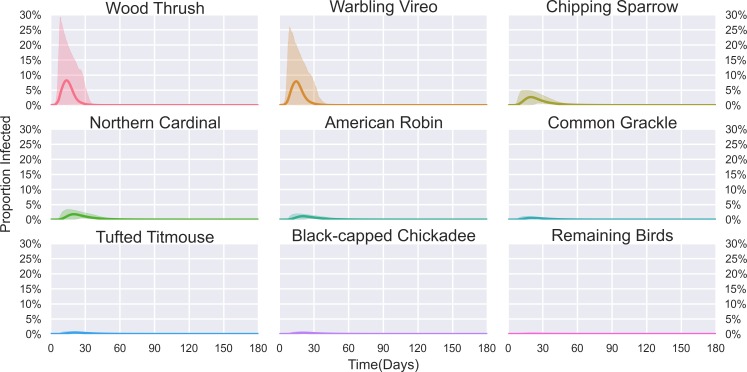
Simulated proportion infected of the selected host species over a 180 day time period beginning April 1, with the shaded region representing the 95% confidence interval of infection.

The eight selected bird species (Wood Thrush, American Robin, Tufted Titmouse, Common Grackle, Chipping Sparrow, Black-capped Chickadee, Northern Cardinal, and Warbling Vireo) were found to have a larger feeding index (Table L in S1 Text) than the remaining birds, indicating these species were fed upon more frequently by *Cs*. *melanura* (Tables 3–6 and C-F in S1 Text). The feeding index was highest for Wood Thrush followed by Warbling Vireo. As a result, both Wood Thrush and Warbling Vireo see early peaks in infections (Fig 4). The infections in Wood Thrush in turn increase the prevalence of infection in *Cs*. *melanura* (Fig C in S1 Text). As a result, the infection rate for some of the remaining bird species, which are less preferred as blood meal hosts, can be seen to increase slightly, allowing EEE virus to persist through the entire season.

## Discussion

Our study examines vector–host interactions of *Cs*. *melanura*, the principal vector of EEE virus in the northeastern U.S., and demonstrates how a relatively limited number of avian hosts may regulate the dynamics of pathogen transmission in complex host communities. We found that Wood Thrush, in particular, may effectively function as a principal reservoir host that serves to amplify EEE virus during the early summer months, whereas less preferred avian hosts facilitate sustained transmission and persistence of virus throughout the remainder of the summer and early fall. These results have broader implications for understanding the perpetuation of vector-borne pathogens in species-rich host communities. The concept of dilution effect has recently been proposed which purports that increases in host diversity may lead to a reduction in disease risk due to the dilution of competent host species [27]. This premise has been applied to tick-borne Lyme disease but its application and relevance to other vector-borne pathogens has been questioned [28]. EEE virus occurs in freshwater swamps where host species diversity is relatively high: in our study sites, we encountered up to 99 avian species. Nevertheless, our model demonstrates that EEE virus may readily amplify in these ecological settings because the mosquito vector *Cs*. *melanura* preferentially feeds on a few virus-competent bird species.

Wood Thrush served as the most frequent host for *Cs*. *melanura* in Madison (28.5%) and Killingworth (26.9%), and was relatively frequent in North Stonington (11.8%) and Chester (4.6%). Similarly, in earlier studies, Wood Thrush was identified as the most frequent host for *Cs*. *melanura* (23.6%) in New York, as the 2nd most frequent (12.5%) in Connecticut, and as a relatively frequent host (5.1%) in Massachusetts [12–14]. Wood Thrush breed in deciduous and mixed forests in the eastern U.S. where there are large trees, moderate understory, shade, and abundant leaf litter for foraging. The breeding range for these birds extends from Manitoba, Ontario, and Nova Scotia in southern Canada to northern Florida, and from the Atlantic coast to the Missouri River and the eastern Great Plains [29]. Wood Thrush was an important source of blood meal later in the season (August-September) where 85.1% (n = 57) of all blood meals were from this bird species in Killingworth, 80.6% (n = 83) in Madison, 80.0% (n = 16) in North Stonington, and 62.6% (n = 10) in Chester (August-October). The intensity of mosquito feeding on this bird species late in the season overlaps with the molt period in adult Wood Thrush that extends from July to early October, a period during which they lose flight feathers, and some individuals drop several primaries over a few days [30]. This extensive molting impairs flight efficiency and makes them remarkably cautious and difficult to observe [30].

Frequent infection of Wood Thrush with EEE virus has been reported. Of the 42 isolations of EEE virus from more than 3,000 birds bled in southern Alabama, there were more from Wood Thrush than any other bird species [31]. In New Jersey, early-season virus isolation from Wood Thrush and a few other bird species has been reported as evidence of a cryptic EEE virus cycle [2]. In Massachusetts, Wood Thrush had the highest EEE virus antibody prevalence rate (26.7%) among 20 avian species examined [16]. In a study conducted in a prominent EEE virus focus surrounding Toad Harbor Swamp in upstate New York, Wood Thrush had antibody prevalence rates of 7.0%, 9.6%, and 50% during 1978, 1979, and 1980, respectively [32]. EEE virus was also isolated from a migrating Wood Thrush in the Mississippi Delta [33].

American Robin served as the second most frequent host for *Cs*. *melanura* in Killingworth (18.5%), Madison (14.1%), and Chester (10.9%), and the third most frequent host in North Stonington (11.8%). Similarly, American Robin served as the most frequent source of blood meal for *Cs*. *melanura* in neighboring Massachusetts (21.7%), and in Connecticut in an earlier study (22.9%), and as the second most frequent host in New York (9.1%) [12–14]. American Robin has also been reported as a frequent host for other mosquito species such as *Culex* spp. throughout the Northeast and other regions of the U.S., highlighting the role of this bird species in the amplification of another avian arbovirus: West Nile virus [10,19,34–36].

American Robin is a common bird species throughout most of North America with permanent and migratory populations. Populations of this avian species inhabit a wide variety of open and forested habitats in urban/suburban and rural settings, riparian forests, early successional forests, and closed canopy forests and woodlands [37–40]. American Robin can occur in large flocks that roost communally in woodland habitats during summer months after nesting ends. Within these roosts, it can be the most prominent tree-roosting bird [41]. The first brood of American Robin emerges in late April through June in southern regions of the Northeast, and in May through early July in northern areas (e.g., northern Maine) providing temporal overlaps with the first generation of *Cs*. *melanura*.

American Robin is a competent amplifying host for EEE virus, and the virus has been isolated from this bird in Massachusetts and New Jersey [2,16,42]. Serosurveys indicate American Robin is frequently exposed to EEE virus throughout the region [2,16, 43–45]. Identification of American Robin as a frequent host in this study, in conjunction with its abundance and other evidence, suggests that this bird species contributes to EEE virus amplification in the region.

Tufted Titmouse was identified as the most frequent host for *Cs*. *melanura* in Chester (22.4%) and a frequent host in Killingworth (15.7%), North Stonington (5.9%), and Madison (5.8%). Our earlier vector-host interaction study in Massachusetts also reported that Tufted Titmouse served as the second most frequently identified host (8.7%) for *Cs*. *melanura* [14]. Tufted Titmouse is common east of the Great Plains in the woodlands of the southeastern, eastern, and midwestern U.S., and in southern Ontario, Canada. Tufted Titmouse prefers deciduous woods or mixed evergreen-deciduous woods, especially moist woodlands found in swamps and river basins, and areas with a dense canopy and many tree species [46]. Tufted Titmouse does not migrate extensively, and remains in residence throughout the winter [47]. Tufted Titmouse was shown to have particularly high antibody prevalence (44.2%) for EEE virus, and specimens of this species were also captured with active viremia in Cape May, New Jersey [2].

The mathematical model we developed is, to our knowledge, the first for EEE virus, a pathogen with many potential hosts, in the northeastern U.S. The most notable result of the model is the dominant role played by Wood Thrush in amplifying EEE virus. Relative to the other bird species, Wood Thrush had a small observed population in the bird counts but a high number of identified blood meals from field-collected mosquitoes. As a result, Wood Thrush had the largest feeding index, and hence played the largest role in the spreading of EEE virus amongst the various bird populations in the model.

We chose to focus on eight bird species due to both their large populations in the bird counts and the large number of blood meals collected from these species. To maintain simplicity in the model, and due to small sample sizes for the remaining bird species, the remaining observed species were combined into a single class. Future work could attempt to identify groups of bird species that are necessary to accurately model EEE virus dynamics. Combining bird species into groups like migratory vs non-migratory could give a clearer understanding of the role of various bird species provided such grouping accurately models EEE virus dynamics. Modeling migratory birds might even allow the data between different sites to be linked, and could potentially be used to track movement from more southerly regions where the virus circulates all year long.

### Potential issues/Model limitations

In order to understand the role that vector feeding preference plays in the amplification of EEE virus, and given the limitations of the collected data, several assumptions were made. Data to estimate the transmission and recovery rates for the host species is limited. As a result, we chose these rates to be the same across the host species. With the assumption that transmission rates do not vary by species, limited sensitivity analysis has shown that species that act as amplifying hosts are insensitive to changes in transmission and recovery rates. If data were available to estimate these parameters separately for each host, then a wide range of substantially different dynamic patterns of infection between host species would be possible.

Pooling the samples over time leads to a model that does not include changes in the mosquito or bird populations over the 180-day model period. If the observed seasonal changes in bird species abundance were incorporated into the model, we expect that overall infection rates amongst the bird species would remain about the same, but the time when infections peak in each species could shift depending on the abundance over time. Further investigations are underway to better understand the potential influence of these factors. We have also assumed that the feeding index values remain constant across the 180-day model period. Wood Thrush may be more susceptible to being bitten during their molting period from late July through August; incorporating this shift in biting preference over time into our model would lead to Wood Thrush becoming infected later in the season than in our current results [30].

Several bird species had comparatively few observed bird counts or identified blood meals. In particular, some bird species in some sample periods had 0 observations in the bird count but a positive number of blood meals, which for our simple estimate would give an infinite feeding index in these periods. As a result, these feeding index values are extremely sensitive to small perturbations in the data. This sensitivity can be seen in the confidence intervals for the feeding index for both Wood Thrush and Warbling Vireo, as both species had relatively small observed bird counts, which led to high coefficients of variation in our Poisson sampling model for bird counts. In order to further examine previously excluded host species, more sophisticated sampling models would be needed.

Despite these limitations, the model provides valuable insight into the role that vector feeding preferences play in the transmission of EEE virus. In particular we notice that observed bird species with relatively small abundances could drive a majority of the infections across all bird species, due to the amplification of the virus in those species early in the season.

In conclusion, we found that *Cs*. *melanura* was exposed to diverse avian communities but preferentially focused feeding on Wood Thrush. The model suggests that this species may play a vital role in supporting EEE virus amplification, subject to confirmation that Wood Thrush is a competent host. *Culiseta melanura* had fed frequently on several other bird species, including American Robin, Tufted Titmouse, Common Grackle, Chipping Sparrow, Black-capped Chickadee, Northern Cardinal, and Warbling Vireo, that were shown to play a less important role in maintaining EEE virus transmission later in the season.

## Supporting Information

S1 Text Table A. EEE virus positive mosquito pools in Chester, Killingworth, Madison and North Stonington, CT, 1996–2014. **Table B.** Number of other engorged mosquitoes collected form Chester, Killingworth, Madison, and North Stonington, CT, May through October, 2010–2011. **Table C.** Number and percentage of avian- and mammalian-derived blood meals identified from *Cs*. *melanura* in Chester, CT, May through October, 2010–2011. (*R.C. = Residency codes: P, permanent resident (found year round in the state); S, summer resident [present in the state during the nesting season]; M, migratory [migrates through the state in spring and/or fall]). **Table D.** Number and percentage of avian- and mammalian-derived blood meals identified from *Cs*. *melanura* in Killingworth, CT, May through October, 2010–2011. (*R.C. = Residency codes: P, permanent resident (found year round in the state); S, summer resident [present in the state during the nesting season]; M, migratory [migrates through the state in spring and/or fall]). **Table E.** Number and percentage of avian- and mammalian-derived blood meals identified from *Cs*. *melanura* in Madison, CT, May through October, 2010–2011. (*R.C. = Residency codes: P, permanent resident (found year round in the state); S, summer resident [present in the state during the nesting season]; M, migratory [migrates through the state in spring and/or fall]). **Table F.** Number and percentage of avian- and mammalian-derived blood meals identified from *Cs*. *melanura* in North Stonington, CT, May through October, 2010–2011. (*R.C. = Residency codes: P, permanent resident (found year round in the state); S, summer resident [present in the state during the nesting season]; M, migratory [migrates through the state in spring and/or fall]). **Table G.** Number and percentage of avian families (N = 37) based on point count data in Chester, Killingworth, Madison, and North Stonington, CT, May through October, 2010–2011 **Table H.** Frequencies of 99 avian species (in descending order from most to least frequently observed) based on point count data in Chester, CT, April through October, 2010–2011 (No. of sites = 7, No. of site visits = 43, comprising 301 point counts.) **Table I.** Frequencies of 66 avian species (in descending order from most to least frequently observed) based on point count data in Killingworth, CT, April through October, 2010–2011 (No. of sites = 3, No of site visits = 44, comprising 132 point counts.) **Table J.** Frequencies of 66 avian species (in descending order from most to least frequently observed) based on point count data in Madison, CT, April through October, 2010–2011 (No. of sites = 4, No. of site visits = 43, comprising 172 point counts.) **Table K.** Frequencies of 68 avian species (in descending order from most to least frequently observed) based on point count data in North Stonington CT, April through October 2011 (No. of sites = 3, No. of site visits = 28, comprising 84 point counts.) **Table L.** Feeding index for each host bird species. Feeding index is the relative likelihood of a blood meal on a given bird species per bird of that species, which indicates the preference of mosquitoes for feeding on different bird species. The feeding index for the named bird species are relative to the remaining, other birds, for which the feeding index was chosen to be 1. **Fig A. Population abundance and peak seasonal activity of *Cs*. *melanura*.** Population abundance and peak seasonal activity of adult female *Cs*. *melanura* in four study sites, Chester, Killingworth, Madison and North Stonington, CT, 2010–2011. **Fig B. Avian point count surveys form.** Status and habitat codes, including observations of nestlings, fledglings, and juvenile birds, and individuals detected outside or flying over the point count circle. **Fig C. Force of infection due to each host species.** The solid line is the median value over the 1000 samples produced, which the shaded region represents the 95% confidence interval. **Modeling. References.**
(DOCX)

## References

[1] Morris CD. 1988 Eastern equine encephalomyelitis Monath TP, ed. The Arboviruses: Epidemiology and Ecology. Boca Raton, FL: CRC Press, 1–20.

[2] Crans WJ, Caccamise DF, McNelly JR. Eastern equine encephalomyelitis virus in relation to the avian community of a coastal cedar swamp. J Med Entomol. 1994; 31: 711–728. 7966175 10.1093/jmedent/31.5.711

[3] Hayes RO. 1981 Eastern and western encephalitis Beran GW, ed. Zoonoses Viral, Section B. Vol. 1 Boca Raton, FL: CRC Handbook Series in Zoonoses, 29–57.

[4] Morris CD, Zimmerman RH. Epizootiology of eastern equine encephalomyelitis virus in upstate New York, USA. III. Population dynamics and vector potential of adult *Culiseta morsitans* (Diptera: Culicidae). J Med Entomol. 1981; 18: 313–316. 7265134 10.1093/jmedent/18.4.313

[5] Scott TW, Weaver SC. Eastern equine encephalomyelitis virus: epidemiology and evolution of mosquito transmission. Adv Virus Res. 1989; 37: 277–328. 2574935 10.1016/s0065-3527(08)60838-6

[6] Howard JJ, Grayson MA, White DJ, Morris CD. Eastern equine encephalitis in New York State. J FL Mosq Cont Assoc. 1994; 65: 1–7.

[7] Armstrong PM, Andreadis TG. Eastern equine encephalitis virus-old enemy, new threat. N Engl J Med. 2013; 368: 1670–1673. 10.1056/NEJMp1213696 23635048

[8] Magnarelli LA. Host feeding patterns of Connecticut mosquitoes (Diptera: Culicidae). Am J Trop Med Hyg. 1977; 26: 547–552. 17310 10.4269/ajtmh.1977.26.547

[9] Morris CD, Zimmerman RH, Edman JD. Epizootiology of eastern equine encephalomyelitis virus in upstate New York, USA. II. Population dynamics and vector potential of adult *Culiseta melanura* (Diptera: Culicidae) in relation to distance from breeding site. J Med Entomol. 1980; 17: 453–465. 6106718 10.1093/jmedent/17.5.453

[10] Apperson CS, Hassan HK, Harrison BA, Savage HM, Aspen SE, Faraji (Farajollahi) A, et al Host-feeding patterns of established and potential mosquito vectors of West Nile virus in the eastern United States. Vector Borne Zoonotic Dis. 2004; 4: 71–82. 15018775 10.1089/153036604773083013 PMC2581457

[11] Hassan HK, Cupp EW, Hill GE, Katholi CR, Klingler K, Unnasch TR. Avian host preference by vectors of eastern equine encephalomyelitis virus. Am J Trop Med Hyg. 2003; 69: 641–647. 14740882

[12] Molaei G, Andreadis TG. Identification of avian- and mammalian-derived bloodmeals in *Aedes vexans* and *Culiseta melanura* (Diptera: Culicidae) and its implication for West Nile virus transmission in Connecticut, USA. J Med Entomol. 2006; 43: 1088–1093. 17017250 10.1603/0022-2585(2006)43[1088:IOAAMB]2.0.CO;2

[13] Molaei G, Oliver J, Andreadis TG, Armstrong PM, Howard JJ. Molecular identification of blood meal sources in *Culiseta melanura* and *Culiseta morsitans* from a focus of eastern equine encephalomyelitis (EEE) virus transmission in New York, USA. Am J Trop Med Hyg. 2006; 75: 1140–1147. 17172382

[14] Molaei G, Andreadis TG, Armstrong PM, Thomas MC, Deschamps T, Cuebas-Incle E, et al Vector-host interactions and epizootiology of eastern equine encephalitis virus in Massachusetts, USA. Vector Borne Zoonotic Dis. 2013; 13: 312–323. 10.1089/vbz.2012.1099 23473221

[15] Howard JJ, Oliver J, Grayson MA. Antibody response of wild birds to natural infection with Alphaviruses. J Med Entomol. 2004; 41: 1090–1103. 15605649 10.1603/0022-2585-41.6.1090

[16] Main AJ, Anderson KS, Maxfield HK, Rosenau B, Oliver C. Duration of Alphavirus neutralizing antibody in naturally infected birds. Am J Trop Med Hyg. 1988; 38: 208–217. 2829638 10.4269/ajtmh.1988.38.208

[17] Morris CD. A structural and operational analysis of diurnal resting shelters for mosquitoes (Diptera: Culicidae). J Med Entomol. 1981; 18: 419–424.

[18] Andreadis TG, Thomas MC, Shepard JJ. 2005 Identification guide to mosquitoes of Connecticut Experiment Station Bulletin 966. The Connecticut Agricultural Experiment Station, New Haven, CT.

[19] Molaei G, Andreadis TG, Armstrong PM, Anderson JF, Vossbrinck CR. Host feeding patterns of *Culex* mosquitoes and West Nile virus transmission, northeastern United States. Emerg Infect Dis. 2006; 12: 468–474. 16704786 10.3201/eid1203.051004 PMC3291451

[20] National Center for Biotechnology Information, The BLAST Sequence Analysis Tool. [http://blast.ncbi.nlm.nih.gov/Blast.cgi?PROGRAM=blastn&PAGE_TYPE=BlastSearch&LINK_LOC=blasthome].

[21] Ralph CJ, Geupel GR, Pyle P, Martin TE, DeSante DF. 1993 Handbook of field methods for monitoring landbirds In: Agriculture USDo, editor. Albany, CA: Forest Service, Pacific Southwest Research Station.

[22] Ralph C, Sauer J, Droege S. 1995 Monitoring bird populations by point counts In: U.S. Department of Agriculture FS, editor. Albany, CA: Pacific Southwest Research Station.

[23] American Ornithologists' Union. 1983 Checklist of North American Birds. 7th edition American Ornithologists' Union, Washington, D.C.

[24] Kay BH, Boreham PFL, Edman JD. Application of the feeding index concept to studies of mosquito host-feeding patterns. Mosq News. 1979; 39: 68–72.

[25] Simpson JE, Hurtado PJ, Medlock J, Molaei G, Andreadis TG, Galvani AP, et al Vector host-feeding preferences drive transmission of multi-host pathogens: West Nile virus as a model system. Proc Biol Sci. 2012; 279: 925–933. 10.1098/rspb.2011.1282 21849315 PMC3259921

[26] Vaidyanathan R, Edman JD, Cooper LA, Scott TW. Vector competence of mosquitoes (Diptera: Culicidae) from Massachusetts for a sympatric isolate of eastern equine encephalomyelitis virus. J Med Entomol. 1997; 34: 346–352. 9151501 10.1093/jmedent/34.3.346

[27] Ostfeld RS, Keesing F. Effects of host diversity on infectious disease. Ann Rev Ecol Evol Syst. 2012; 43: 157–182.

[28] Randolph SE, Dobson AD. Pangloss revisited: a critique of the dilution effect and the biodiversity-buffers-disease paradigm. Parasit. 2012; 139: 847–863. 10.1017/S0031182012000200 22336330

[29] Bull J, Farrand J Jr. 1987 Audubon Society Field Guide to North American Birds: Eastern Region. New York: Alfred A. Knopf pp. 666–667. ISBN 0-394-41405-5.

[30] Vega Rivera JH, McShea WJ, Rappole JH, Haas CA. Pattern and chronology of prebasic molt for the Wood Thrush and its relation to reproduction and migration departure. The Wilson Bulletin. 1998; 110: 384–392.

[31] Stamm D. Arbovirus studies in birds in South Alabama 1959–1960. Am J Epidemiol. 1968; 67: 127–137. 10.1093/oxfordjournals.aje.a120793 5637863

[32] Emord DE, Morris CD. Epizootiology of eastern equine encephalomyelitis in upstate, New York, USA. VI. Antibody prevalence in wild birds during an interepizootic period. J Med Entomol. 1984; 21: 395–404. 6092637 10.1093/jmedent/21.4.395

[33] Calisher CH, Maness KS, Lord RD, Coleman PH. Identification of two South American strains of eastern equine encephalomyelitis virus from migrant birds captured on the Mississippi delta. Am J Epidemiol. 1971; 94: 172–178. 5568603 10.1093/oxfordjournals.aje.a121309

[34] Apperson CS, Harrison BA, Unnasch TR, Hassan HK, Irby WS, Savage HM, et al Host feeding habits of Culex and other mosquitoes (Diptera: Culicidae) in the Borough of Queens in New York City, with characters and techniques for identification of Culex mosquitoes. J Med Entomol. 2002; 39: 777–785. 12349862 10.1603/0022-2585-39.5.777

[35] Savage HM, Aggarwal D, Apperson CS, Katholi CR, Gordon E, Hassan HK, et al Host choice and West Nile virus infection rates in blood-fed mosquitoes, including members of the *Culex pipiens* complex, from Memphis and Shelby County, Tennessee, 2002–2003. Vector Borne Zoonotic Dis. 2007; 7: 365–386. 17767413 10.1089/vbz.2006.0602 PMC2580743

[36] Hamer GL, Kitron U, Goldberg TL, Brawn JD, Loss SR, Ruiz MO, et al Host selection by *Culex pipiens* mosquitoes and West Nile Virus amplification. Am J Trop Med Hyg. 2009; 80: 268–278. 19190226

[37] Martin K. 1973. Breeding density and reproductive success of Robins in relation to habitat structure on logged areas of Vancouver Island, British Columbia. MS Thesis. University of Alberta, Edmonton, Canada.

[38] Hutto RL. The composition of bird communities following stand-replacement fires in northern Rocky Mountain conifer forests. Conserv Biol. 1995; 9: 1–19. 10.1046/j.1523-1739.1995.9051033.x-i1 34261259

[39] Sallabanks R. 1995. Effects of wildfire on breeding bird communities in coniferous forests of northwestern Oregon. Available through Blue Mountains Natural Resources Institute online at http://www.fs.fed.us/pnw/mdr/past/bmnri/publications/abstract/sallabanks01.shtml.

[40] United States Environmental Protection Agency [http://goo.gl/1GIDB]http://www.epa.gov/region1/ge/thesite/restofriver/reports/final_era/B%20-%20Focus%20Species%20Profiles/EcoRiskProfile_american_robin.pdf].

[41] Poole, A. (Editor). 2005. The birds of North America online: Cornell Laboratory of Ornithology, Ithaca, NY (http://bna.birds.cornell.edu/BNA/ 2005).

[42] Komar N, Dohm DJ, Turell MJ, Spielman A. Eastern equine encephalitis virus in birds: relative competence of European starlings (*Sturnus vulgaris*). Am J Trop Med Hyg. 1999; 60: 387–391. 10466964 10.4269/ajtmh.1999.60.387

[43] Dalrymple JM, Young OP, Eldridge BF, Russell PK. Ecology of arboviruses in a Maryland freshwater swamp. III. Vertebrate hosts. Am J Epidemiol. 1972; 96: 129–140. 4403123 10.1093/oxfordjournals.aje.a121439

[44] Bast TF, Whitney E, Benach JL. Considerations on the ecology of several arboviruses in eastern Long Island. Am J Trop Med Hyg. 1973; 22: 109–115. 4684881 10.4269/ajtmh.1973.22.109

[45] Morris CD, Caines AR, Woodall JP, Bast TF. Eastern equine encephalomyelitis in upstate New York, 1972–1974. Am J Trop Med Hyg. 1975; 24: 986–991. 919 10.4269/ajtmh.1975.24.986

[46] The Cornel Laboratory of Ornithology, All About Birds. [http://www.allaboutbirds.org/guide/Tufted_Titmouse/lifehistory].

[47] Singh A, Frazier A. https://pages.vassar.edu/sensoryecology/tufted-titmouse-baeolophus-bicolor-general-biology/, Posted on May 3, 2015 by absingh.

