# Supplementary material for: Dynamics of Vector-Host Interactions in Avian Communities in Four Eastern Equine Encephalitis Virus Foci in the Northeastern U.S
Source: PLoS Negl Trop Dis. 2016 Jan 11;10(1):e0004347. doi: 10.1371/journal.pntd.0004347 (PMC4713425; doi:10.1371/journal.pntd.0004347)
Supplement: S1 Text — Table B. Number of other engorged mosquitoes collected form Chester, Killingworth, Madison, and North Stonington, CT, May through October, 2010–2011. Table C. Number and percentage of avian- and mammalian-derived blood meals identified from Cs. melanura in Chester, CT, May through October, 2010–2011. (*R.C. = Residency codes: P, permanent resident (found year round in the state); S, summer resident [present in the state during the nesting season]; M, migratory [migrates through the state in spring and/or fall]). Table D. Number and percentage of avian- and mammalian-derived blood meals identified from Cs. melanura in Killingworth, CT, May through October, 2010–2011. (*R.C. = Residency codes: P, permanent resident (found year round in the state); S, summer resident [present in the state during the nesting season]; M, migratory [migrates through the state in spring and/or fall]). Table E. Number and percentage of avian- and mammalian-derived blood meals identified from Cs. melanura in Madison, CT, May through October, 2010–2011. (*R.C. = Residency codes: P, permanent resident (found year round in the state); S, summer resident [present in the state during the nesting season]; M, migratory [migrates through the state in spring and/or fall]). Table F. Number and percentage of avian- and mammalian-derived blood meals identified from Cs. melanura in North Stonington, CT, May through October, 2010–2011. (*R.C. = Residency codes: P, permanent resident (found year round in the state); S, summer resident [present in the state during the nesting season]; M, migratory [migrates through the state in spring and/or fall]). Table G. Number and percentage of avian families (N = 37) based on point count data in Chester, Killingworth, Madison, and North Stonington, CT, May through October, 2010–2011 Table H. Frequencies of 99 avian species (in descending order from most to least frequently observed) based on point count data in Chester, CT, April through October, 2010–2011 (No. of sit [file pntd.0004347.s001.docx]

**Table A.** EEE virus positive mosquito pools in Chester, Killingworth, Madison and North Stonington, CT, 1996-2014

| **Mosquito Species** | **No. of Positive Mosquito Pools** | | | | |
| --- | --- | --- | --- | --- | --- |
|  | **Chester** | **Killingworth** | **Madison** | **North Stonington** | **Total (%)** |
| *Culiseta melanura* | 26 | 9 | 11 | 28 | 74 (64.3) |
| *Ochlerotatus canadensis* | 10 | 2 |  | 1 | 13 (11.3) |
| *Ochlerotatus trivittatus* |  |  |  | 6 | 6 (5.2) |
| *Aedes cinereus* | 1 | 2 |  | 2 | 5 (4.3) |
| *Uranotaenia sapphirina* | 2 |  | 1 | 2 | 5 (4.3) |
| *Aedes vexans* | 1 |  |  | 3 | 3 (2.6) |
| *Anopheles punctipennis* | 2 |  |  | 1 | 3 (2.6) |
| *Culex pipiens* |  |  |  | 2 | 2 (1.7) |
| *Anopheles quadrimaculatus* |  |  |  | 1 | 1 (0.9) |
| *Anopheles walkeri* |  |  |  | 1 | 1 (0.9) |
| *Culex restuans* |  |  | 1 |  | 1 (0.9) |
| *Ochlerotatus triseriatus* |  |  |  | 1 | 1 (0.9) |
| Total | 42 (36.5) | 13 (11.3) | 13 (11.3) | 47 (40.9) | 115 |

From 1996 to 2014, a total of 115 mosquito pools of 12 mosquito species have been tested positive for EEE virus by cell culture and RT-PCR. *Cs. melanura* comprised the majority of positive pools (N = 74, 64.3%), followed by *Ochlerotatus canadensis* (N = 13, 11.3%), *Ochlerotatus trivittatus* (N = 6, 5.2%), *Aedes cinereus* (N = 5, 4.3%), *Uranotaenia sapphirina* (N = 5, 4.3%), and 7 other species. North Stonington had the greatest number of positive pools (N = 47, 40.9%), followed by Chester (N = 42, 36.5%), Killingworth (N = 13, 11.3%), and Madison (N = 13, 11.3%). Majority of the positive pools were identified during 2009 (N = 56, 48.7%), which contributed to the rationales behind the initiation of the present study during 2010 - 2011. In 2010, four mosquito pools tested positive in mosquitoes from North Stonington but none from the other sites. Interestingly, positive mosquito pools were identified in North Stonington in 8 years of the nearly two decades during which active mosquito surveillance has been conducted for EEEV in Connecticut.

**Table B.** Number of other engorged mosquitoes collected form Chester,

Killingworth, Madison, and North Stonington, CT, May through October,

2010 – 2011

| **Mosquito Species** | **No.** |
| --- | --- |
| *Ochlerotatus thibaulti* | 202 |
| *Culiseta morsitans* | 54 |
| *Anopheles punctipennis* | 48 |
| *Anopheles quadrimaculatus* | 25 |
| *Culex territans* | 19 |
| *Aedes cinereus* | 6 |
| *Coquillettidia perturbans* | 6 |
| *Culex restuans* | 4 |
| *Ochlerotatus abserratus* | 3 |
| *Culex pipiens* | 2 |
| *Ochlerotatus canadensis* | 2 |
| *Ochlerotatus Stimulans* | 1 |

In addition to *Cs. melanura*, 372 engorged specimens of 12 species in the genera of *Aedes, Anopheles, Coquillettidia, Culex, Culiseta,* and *Ochlerotatus* were collected and blood meal analyses conducted. However, because of the focus of the present study on *Cs. melanura*, the principal mosquito vector of EEE virus, results of these analyses are not presented.

**Table C.** Number and percentage of avian- and mammalian-derived blood meals

identified from *Culiseta melanura* in Chester, CT, May through October, 2010 –

2011. ^*^R.C. = Residency codes: P, permanent resident (found year round in the state);

S, summer resident [present in the state during the nesting season]; T, transient.

| **Vertebrate Host** | **Scientific Name** | **R. C.^*^** | **May**  **No. (%)** | **June**  **No. (%)** | **July**  **No. (%)** | **Aug**  **No. (%)** | **Sept**  **No. (%)** | **Oct**  **No. (%)** | **Total** |
| --- | --- | --- | --- | --- | --- | --- | --- | --- | --- |
|  |  |  |  |  |  |  |  |  |  |
| **Avian** |  |  |  |  |  |  |  |  |  |
| Tufted Titmouse | *Baeolophus bicolor* | P | 1 (100) | 7 (6.5) | 11(14.3) | 29 (29.6) | 26 (44.1) | 4 (66.7) | 78 |
| American Robin | *Turdus migratorius* | P, T |  | 15 (14.0) | 10 (13.0) | 10 (10.2) | 3 (5.1) |  | 38 |
| Common Grackle | *Quiscalus quiscula* | P, T |  | 22 (20.6) | 1 (1.3) | 4 (4.1) | 1 (1.7) |  | 28 |
| Warbling Vireo | *Vireo gilvus* | S |  | 7 (6.5) | 16 (20.8) |  |  |  | 23 |
| Red-eyed Vireo | *Vireo olivaceus* | S |  | 5 (4.7) |  | 10 (10.2) | 8 (13.6) |  | 23 |
| Black-capped Chickadee | *Poecile atricapillus* | P |  | 6 (5.6) | 7 (9.1) | 8 (8.2) |  |  | 21 |
| Wood Thrush | *Hylocichla mustelina* | S |  | 2 (1.9) | 4 (5.2) | 3 (3.1) | 5 (8.5) | 2 (33.3) | 16 |
| Chipping Sparrow | *Spizella passerina* | S |  | 4 (3.7) | 3 (3.9) | 5 (5.1) | 2 (3.4) |  | 14 |
| Yellow-throated Vireo | *Vireo flavifrons* | S |  | 4 (3.7) |  | 2 (2.0) | 6 (10.2) |  | 12 |
| Northern Cardinal | *Cardinalis cardinalis* | P |  | 4 (3.7) |  | 4 (4.1) | 1 (1.7) |  | 9 |
| Scarlet Tanager | *Piranga olivacea* | S |  | 3 (2.8) | 5 (6.5) |  | 1 (1.7) |  | 9 |
| Ovenbird | *Seiurus aurocapilla* | S |  | 4 (3.7) | 3 (3.9) | 1 (1.0) |  |  | 8 |
| Blue-gray Gnatcatcher | *Polioptila caerulea* | S |  | 2 (1.9) | 1 (1.3) | 3 (3.1) |  |  | 6 |
| Gray Catbird | *Dumetella carolinensis* | S |  | 4 (3.7) | 2 (2.6) |  |  |  | 6 |
| American Goldfinch | *Spinus tristis* | P |  |  |  | 5 (5.1) |  |  | 5 |
| Brown-headed Cowbird | *Molothrus ater* | P, T |  | 3 (2.8) | 1 (1.3) | 1 (1.0) |  |  | 5 |
| Mourning Dove | *Zenaida macroura* | P |  |  | 1 (1.3) | 4 (4.1) |  |  | 5 |
| Baltimore Oriole | *Icterus galbula* | S |  | 1 (0.9) | 3 (3.9) |  |  |  | 4 |
| Rose-breasted Grosbeak | *Pheucticus ludovicianus* | S |  | 1 (0.9) | 1(1.3) |  | 2 (3.4) |  | 4 |
| Common Yellowthroat | *Geothlypis trichas* | S |  | 1 (0.9) |  | 1 (1.0) | 1 (1.7) |  | 3 |
| European Starling | *Sturnus vulgaris* | P |  | 1 (0.9) | 1 (1.3) |  | 1 (1.7) |  | 3 |
| Red-winged Blackbird | *Agelaius phoeniceus* | P, T |  | 1 (0.9) | 2 (2.6) |  |  |  | 3 |
| American Redstart | *Setophaga ruticilla* | S |  |  | 1 (1.3) | 1 (1.0) |  |  | 2 |
| Great Horned Owl | *Bubo virginianus* | P |  |  |  | 2 (2.0) |  |  | 2 |
| Yellow-billed Cuckoo | *Coccyzus americanus* | S |  |  | 1 (1.3) | 1 (1.0) |  |  | 2 |
| American Crow | *Corvus brachyrhynchos* | P |  | 1 (0.9) |  |  |  |  | 1 |
| American Woodcock | *Scolopax minor* | S |  | 1 (0.9) |  |  |  |  | 1 |
| Barn Swallow | *Hirundo rustica* | S |  |  | 1 (1.3) |  |  |  | 1 |
| Black-and-white Warbler | *Mniotilta varia* | S |  |  | 1 (1.3) |  |  |  | 1 |
| Cedar Waxwing | *Bombycilla cedrorum* | P |  | 1 (0.9) |  |  |  |  | 1 |
| Eastern Towhee | *Pipilo erythrophthalmus* | S |  |  | 1 (1.3) |  |  |  | 1 |
| Eastern Wood-Pewee | *Contopus virens* | S |  |  |  | 1 (1.0) |  |  | 1 |
| Great Crested Flycatcher | *Myiarchus crinitus* | S |  |  |  | 1 (1.0) |  |  | 1 |
| House Wren | *Troglodytes aedon* | S |  |  |  |  | 1 (1.7) |  | 1 |
| Indigo Bunting | *Passerina cyanea* | S |  | 1 (0.9) |  |  |  |  | 1 |
| Swamp Sparrow | *Melospiza georgiana* | S |  |  |  |  | 1 (1.7) |  | 1 |
| Veery | *Catharus fuscescens* | S |  | 1 (0.9) |  |  |  |  | 1 |
| Wood Duck | *Aix sponsa* | S |  |  |  | 1 (1.0) |  |  | 1 |
| Worm-eating Warbler | *Helmitheros vermivorum* | S |  |  |  | 1 (1.0) |  |  | 1 |
| Yellow-rumped Warbler | *Setophaga coronata* | P, T |  | 1 (0.9) |  |  |  |  | 1 |
| **Mammalian** |  |  |  |  |  |  |  |  |  |
| White-tailed Deer | *Odocoileus virginianus* | P |  | 4 (3.7) |  |  |  |  | 4 |
| Total |  |  | 1 | 107 | 77 | 98 | 59 | 6 | 348 |

**Table D.** Number and percentage of avian- and mammalian-derived blood meals

identified from *Culiseta melanura* in Killingworth, CT, May through October,

2010 – 2011. ^*^R.C. = Residency codes: P, permanent resident (found year round in the state); S, summer resident [present in the state during the nesting season]; T, transient.

| **Vertebrate Host** | **Scientific Name** | **R. C.^*^** | **May**  **No. (%)** | **June**  **No. (%)** | **July**  **No. (%)** | **Aug**  **No. (%)** | **Sept**  **No. (%)** | **Oct**  **No. (%)** | **Total** |
| --- | --- | --- | --- | --- | --- | --- | --- | --- | --- |
| **Avian** |  |  |  |  |  |  |  |  |  |
| Wood Thrush | *Hylocichla mustelina* | S | 1 (6.3) | 3 (8.1) | 6 (9.7) | 45 (46.9) | 12 (42.9) |  | 67 |
| American Robin | *Turdus migratorius* | P, T | 6 (37.5) | 15 (40.5) | 13 (21.0) | 8 (8.3) | 2 (7.1) | 2 (20.0) | 46 |
| Tufted Titmouse | *Baeolophus bicolor* | P | 1 (6.3) | 7 (18.9) | 19 (30.6) | 8 (8.3) | 4 (14.3) |  | 39 |
| Black-capped Chickadee | *Poecile atricapillus* | P | 1 (6.3) | 4 (10.8) | 7 (11.3) | 3 (3.1) |  | 1 (10.0) | 16 |
| Northern Cardinal | *Cardinalis cardinalis* | P |  | 1 (2.7) | 3 (4.8) | 5 (5.2) | 1 (3.6) | 2 (20.0) | 12 |
| Common Grackle | *Quiscalus quiscula* | P, T |  | 2 (5.4) | 3 (4.8) | 4 (4.2) | 1 (3.6) | 1 (10.0) | 11 |
| Scarlet Tanager | *Piranga olivacea* | S | 3 (18.8) | 1 (2.7) | 1 (1.6) | 5 (5.2) |  |  | 10 |
| Chipping Sparrow | *Spizella passerina* | S | 1 (6.3) |  | 2 (3.2) | 4 (4.2) | 1 (3.6) | 1 (10.0) | 9 |
| Gray Catbird | *Dumetella carolinensis* | S |  |  | 2 (3.2) | 1 (1.0) | 1 (3.6) |  | 4 |
| Ovenbird | *Seiurus aurocapilla* | S | 1 (6.3) |  |  | 2 (2.1) | 1 (3.6) |  | 4 |
| House Sparrow | *Passer domesticus* | P |  | 1 (2.7) | 1 (1.6) | 1 (1.0) |  |  | 3 |
| Blue-gray Gnatcatcher | *Polioptila caerulea* | S |  |  |  | 2 (2.1) |  |  | 2 |
| Pine Warbler | *Setophaga pinus* | S |  |  |  | 2 (2.1) |  |  | 2 |
| Swamp Sparrow | *Melospiza georgiana* | S | 1 (6.3) | 1 (2.7) |  |  |  |  | 2 |
| Acadian Flycatcher | *Empidonax virescens* | S |  |  |  | 1 (100) |  |  | 1 |
| Red-bellied Woodpecker | *Melanerpes carolinus* | P |  |  |  |  | 1 (3.6) |  | 1 |
| American Goldfinch | *Spinus tristis* | P |  |  |  |  |  | 1 (10.0) | 1 |
| Baltimore Oriole | *Icterus galbula* | S |  |  | 1 (1.6) |  |  |  | 1 |
| Black-and-white Warbler | *Mniotilta varia* | S |  |  |  |  | 1 (3.6) |  | 1 |
| Blue-headed Vireo | *Vireo solitarius* | S |  |  |  |  | 1 (3.6) |  | 1 |
| Brown-headed Cowbird | *Molothrus ater* | P, T |  |  | 1 (1.6) |  |  |  | 1 |
| Common Yellowthroat | *Geothlypis trichas* | S |  |  |  | 1 (1.0) |  |  | 1 |
| Eastern Bluebird | *Sialia sialis* | P, T |  | 1 (2.7) |  |  |  |  | 1 |
| Green Heron | *Butorides virescens* | S |  |  |  | 1 (1.0) |  |  | 1 |
| Hermit Thrush | *Catharus guttatus* | S | 1 (6.3) |  |  |  |  |  | 1 |
| House Wren | *Troglodytes aedon* | S |  |  | 1 (1.6) |  |  |  | 1 |
| Mourning Dove | *Zenaida macroura* | P |  | 1 (2.7) |  |  |  |  | 1 |
| Red-winged Blackbird | *Agelaius phoeniceus* | P, T |  |  |  | 1 (1.0) |  |  | 1 |
| Rose-breasted Grosbeak | *Pheucticus ludovicianus* | S |  |  |  | 1 (1.0) |  |  | 1 |
| Savannah Sparrow | *Passerculus sandwichensis* | S |  |  |  |  | 1 (3.6) |  | 1 |
| Veery | *Catharus fuscescens* | S |  |  |  | 1 (1.0) |  |  | 1 |
| Warbling Vireo | *Vireo gilvus* | S |  |  | 1 (1.6) |  |  |  | 1 |
| White-throated Sparrow | *Zonotrichia albicollis* | W, T |  |  |  |  |  | 1 (10.0) | 1 |
| Yellow-billed Cuckoo | *Coccyzus americanus* | S |  |  |  |  | 1 (3.6) |  | 1 |
| Yellow-throated Vireo | *Vireo flavifrons* | S |  |  | 1 (1.6) |  |  |  | 1 |
| **Mammalian** |  |  |  |  |  |  |  |  |  |
| White-tailed Deer | *Odocoileus virginianus* | P |  |  |  |  |  | 1 (10.0) | 1 |
| Total | |  | 16 | 37 | 62 | 96 | 28 | 10 | 249 |

**Table E.** Number and percentage of avian- and mammalian-derived blood meals identified

from *Culiseta melanura* in Madison, CT, May through October, 2010 – 2011. ^*^R.C. =

Residency codes: P, permanent resident (found year round in the state); S, summer resident

[present in the state during the nesting season]; T, transient.

| **Vertebrate Host** | **Scientific Name** | **R. C.^*^** | **May**  **No. (%)** | **June**  **No. (%)** | **July**  **No. (%)** | **Aug**  **No. (%)** | **Sept**  **No. (%)** | **Oct**  **No. (%)** | **Total** |  |
| --- | --- | --- | --- | --- | --- | --- | --- | --- | --- | --- |
| **Avian** |  |  |  |  |  |  |  |  |  |  |
| Wood Thrush | *Hylocichla mustelina* | S |  | 11 (11.7) | 9 (13.8) | 56 (40.3) | 27 (52.9) |  | 103 |  |
| American Robin | *Turdus migratorius* | P, T | 2 (28.6) | 17 (18.1) | 20 (30.8) | 10 (7.2) | 2 (3.9) |  | 51 |  |
| Common Grackle | *Quiscalus quiscula* | P, T |  | 9 (9.6) | 3 (4.6) | 17 (12.2) | 2 (3.9) |  | 31 |  |
| Tufted Titmouse | *Baeolophus bicolor* | P | 1 (14.3) | 6 (6.4) | 4 (6.2) | 9 (6.5) | 1 (2.0) |  | 21 |  |
| Black-capped Chickadee | *Poecile atricapillus* | P |  | 8 (8.5) | 3 (4.6) | 5 (3.6) | 2 (3.9) |  | 18 |  |
| Northern Cardinal | *Cardinalis cardinalis* | P |  | 7 (7.4) | 3 (4.6) | 3 (2.2) | 3 (5.9) | 1 (20.0) | 17 |  |
| Scarlet Tanager | *Piranga olivacea* | S | 1 (14.3) | 7 (7.4) |  | 8 (5.8) | 1 (2.0) |  | 17 |  |
| Chipping Sparrow | *Spizella passerina* | S |  | 7 (7.4) | 4 (6.2) | 4 (2.9) |  | 1 (20.0) | 16 |  |
| Red-eyed Vireo | *Vireo olivaceus* | S | 1 (14.3) | 3 (3.2) | 5 (7.7) | 4 (2.9) |  |  | 13 |  |
| Gray Catbird | *Dumetella carolinensis* | S |  | 4 (4.3) | 2 (3.1) |  |  | 1 (20.0) | 7 |  |
| Rose-breasted Grosbeak | *Pheucticus ludovicianus* | S | 1 (14.3) | 1 (1.1) |  | 3 (2.2) | 1 (2.0) |  | 6 |  |
| European Starling | *Sturnus vulgaris* | P | 1 (14.3) |  | 1 (1.5) | 1 (0.7) | 1 (2.0) | 1 (20.0) | 5 |  |
| Ovenbird | *Seiurus aurocapilla* | S |  | 2 (2.1) |  | 2 (1.4) | 1 (2.0) |  | 5 |  |
| Baltimore Oriole | *Icterus galbula* | S |  | 1 (1.1) |  | 3 (2.2) |  |  | 4 |  |
| Brown-headed Cowbird | *Molothrus ater* | P, T |  | 4 (4.3) |  |  |  |  | 4 |  |
| House Wren | *Troglodytes aedon* | S |  |  |  | 3 (2.2) |  |  | 3 |  |
| Yellow-throated Vireo | *Vireo flavifrons* | S |  |  | 1 (1.5) |  | 2 (3.9) |  | 3 |  |
| American Redstart | *Setophaga ruticilla* | S |  |  |  |  | 2 (3.9) |  | 2 |  |
| Black-and-white Warbler | *Mniotilta varia* | S |  | 2 (2.1) |  |  |  |  | 2 |  |
| Eastern Towhee | *Pipilo erythrophthalmus* | S |  | 1 (1.1) |  | 1 (0.7) |  |  | 2 |  |
| Hermit Thrush | *Catharus guttatus* | S |  | 1 (1.1) |  |  | 1 (2.0) |  | 2 |  |
| House Finch | *Haemorhous mexicanus* | P |  |  |  | 2 (1.4) |  |  | 2 |  |
| Indigo Bunting | *Passerina cyanea* | S |  | 1 (1.1) | 1 (1.5) |  |  |  | 2 |  |
| Magnolia Warbler | *Setophaga magnolia* | S |  |  |  | 1 (0.7) | 1 (2.0) |  | 2 |  |
| Red-tailed Hawk | *Buteo jamaicensis* | P |  |  |  | 2 (1.4) |  |  | 2 |  |
| Red-winged Blackbird | *Agelaius phoeniceus* | P, T |  |  | 1 (1.5) | 1 (0.7) |  |  | 2 |  |
| Veery | *Catharus fuscescens* | S |  |  |  |  | 2 (3.9) |  | 2 |  |
| Warbling Vireo | *Vireo gilvus* | S |  |  | 2 (3.1) |  |  |  | 2 |  |
| Acadian Flycatcher | *Empidonax virescens* | S |  |  |  | 1 (0.7) |  |  | 1 |  |
| Barn Swallow | *Hirundo rustica* | S |  |  | 1 (1.5) |  |  |  | 1 |  |
| Blackburnian Warbler | *Setophaga fusca* | S |  |  |  |  | 1 (2.0) |  | 1 |  |
| Blue-winged Warbler | *Vermivora cyanoptera* | S |  |  | 1 (1.5) |  |  |  | 1 |  |
| Cedar Waxwing | *Bombycilla cedrorum* | P |  | 1 (1.1) |  |  |  |  | 1 |  |
| Common Yellowthroat | *Geothlypis trichas* | S |  |  |  | 1 (0.7) |  |  | 1 |  |
| Northern Waterthrush | *Parkesia noveboracensis* | S |  | 1 (1.1) |  |  |  |  | 1 |  |
| Pine Warbler | *Setophaga pinus* | S |  |  |  | 1 (0.7) |  |  | 1 |  |
| Song Sparrow | *Melospiza melodia* | P |  |  |  | 1 (0.7) |  |  | 1 |  |
| Swainson's Thrush | *Catharus ustulatus* | T |  |  |  |  |  | 1 (20.0) | 1 |  |
| Wild Turkey | *Meleagris gallopavo* | P |  |  | 1 (1.5) |  |  |  | 1 |  |
| Worm-eating Warbler | *Helmitheros vermivorum* | S |  |  | 1 (1.5) |  |  |  | 1 |  |
| Yellow-billed Cuckoo | *Coccyzus americanus* | S |  |  | 1 (1.5) |  |  |  | 1 |  |
| Yellow-rumped Warbler | *Setophaga coronata* | P, T |  |  | 1 (1.5) |  |  |  | 1 |  |
| **Mammalian** |  |  |  |  |  |  |  |  |  |  |
| Sheep | *Ovis aries* |  |  |  |  |  | 1 (2.0) |  | 1 |  |
| Total |  |  | 7 | 94 | 65 | 139 | 51 | 5 | 361 |  |
|  | | | | | | | | | | |

**Table F.** Number and percentage of avian- and mammalian-derived blood meals identified

from *Culiseta melanura* in North Stonington, CT, May through October, 2010 – 2011.

^*^R.C. = Residency codes: P, permanent resident (found year round in the state); S, summer

resident [present in the state during the nesting season]; T, transient.

| **Vertebrate Host** | **Scientific Name** | **R. C.^*^** | **May**  **No. (%)** | **June**  **No. (%)** | **July**  **No. (%)** | **Aug**  **No. (%)** | **Sept**  **No. (%)** | **Oct**  **No. (%)** | **Total** |
| --- | --- | --- | --- | --- | --- | --- | --- | --- | --- |
| **Avian** |  |  |  |  |  |  |  |  |  |
| Chipping Sparrow | *Spizella passerina* | S |  | 7 (22.6) | 6 (13.0) | 7 (12.7) | 2 (6.9) |  | 22 |
| Northern Cardinal | *Cardinalis cardinalis* | P |  | 1 (3.2) | 4 (8.7) | 11 (20.0) | 5 (17.2) |  | 21 |
| American Robin | *Turdus migratorius* | P, T |  | 5 (16.1) | 5 (10.9) | 7 (12.7) | 3 (10.3) |  | 20 |
| Wood Thrush | *Hylocichla mustelina* | S |  |  | 4 (8.7) | 8 (14.5) | 8 (27.6) |  | 20 |
| Tufted Titmouse | *Baeolophus bicolor* | P |  | 4 (12.9) | 5 (10.9) |  | 1 (3.4) |  | 10 |
| Common Grackle | *Quiscalus quiscula* | P, T |  | 2 (6.5) | 1 (2.2) | 4 (7.3) |  |  | 7 |
| Red-eyed Vireo | *Vireo olivaceus* | S |  | 2 (6.5) | 3 (6.5) | 1 (1.8) |  |  | 6 |
| Yellow-billed Cuckoo | *Coccyzus americanus* | S | 2 (40.0) |  | 3 (6.5) | 1 (1.8) |  |  | 6 |
| Black-capped Chickadee | *Poecile atricapillus* | P |  | 1 (3.2) | 2 (4.3) | 1 (1.8) |  | 1 (33.3) | 5 |
| Rose-breasted Grosbeak | *Pheucticus ludovicianus* | S |  |  | 2 (4.3) | 3 (5.5) |  |  | 5 |
| Scarlet Tanager | *Piranga olivacea* | S |  | 2 (6.5) | 2 (4.3) |  | 1 (3.4) |  | 5 |
| Gray Catbird | *Dumetella carolinensis* | S | 1 (20.0) | 1 (3.2) |  | 2 (3.6) |  |  | 4 |
| Pine Warbler | *Setophaga pinus* | S |  |  |  | 3 (5.5) | 1 (3.4) |  | 4 |
| Veery | *Catharus fuscescens* | S |  | 1 (3.2) | 2 (4.3) |  | 1 (3.4) |  | 4 |
| Warbling Vireo | *Vireo gilvus* | S |  |  | 3 (6.5) | 1 (1.8) |  |  | 4 |
| House Wren | *Troglodytes aedon* | S |  |  | 1 (2.2) | 1 (1.8) | 1 (3.4) |  | 3 |
| Indigo Bunting | *Passerina cyanea* | S |  |  |  | 2 (3.6) | 1 (3.4) |  | 3 |
| Ovenbird | *Seiurus aurocapilla* | S |  | 2 (6.5) | 1 (2.2) |  |  |  | 3 |
| Brown-headed Cowbird | *Molothrus ater* | P, T |  | 1 (3.2) | 1 (2.2) |  |  |  | 2 |
| Cooper's Hawk | *Accipiter cooperii* | P, T |  |  | 1 (2.2) |  | 1 (3.4) |  | 2 |
| American Redstart | *Setophaga ruticilla* | S |  |  |  |  | 1 (3.4) |  | 1 |
| Baltimore Oriole | *Icterus galbula* | S |  | 1 (3.2) |  |  |  |  | 1 |
| Black-billed Cuckoo | *Coccyzus erythropthalmus* | S | 1 (20.0) |  |  |  |  |  | 1 |
| Common Yellowthroat | *Geothlypis trichas* | S |  |  |  | 1 (1.8) |  |  | 1 |
| Dark-eyed Junco | *Junco hyemalis* | W |  |  |  |  |  | 1 (33.3) | 1 |
| Grasshopper Sparrow | *Ammodramus savannarum* | S |  | 1 (3.2) |  |  |  |  | 1 |
| Mourning Dove | *Zenaida macroura* | P | 1 (20.0) |  |  |  |  |  | 1 |
| Red-winged Blackbird | *Agelaius phoeniceus* | P, T |  |  |  | 1 (1.8) |  |  | 1 |
| Sora | *Porzana carolina* | S |  |  |  |  | 1 (3.4) |  | 1 |
| Swamp Sparrow | *Melospiza georgiana* | P, T |  |  |  |  |  | 1 (33.3) | 1 |
| Tree Swallow | *Tachycineta bicolor* | S |  |  |  |  | 1 (3.4) |  | 1 |
| Yellow-rumped Warbler | *Setophaga coronata* | P, T |  |  |  | 1 (1.8) |  |  | 1 |
| **Mammalian** |  |  |  |  |  |  |  |  |  |
| Eastern Gray Squirrel | *Sciurus carolinensis* |  |  |  |  |  | 1 (3.4) |  | 1 |
| Total |  |  | 5 | 31 | 46 | 55 | 29 | 3 | 169 |
|  | | | | | | | | | |

**Table G.** Number and percentage of avian families (N=37) based on point count data in Chester, Killingworth, Madison, and North Stonington, CT, May through October, 2010 – 2011

| **Order/Family** | **Chester** | **Killingworth** | **Madison** | **North Stonington** | **Total** | **%** |  |  |
| --- | --- | --- | --- | --- | --- | --- | --- | --- |
| **Passeriformes** | |  |  |  |  |  |  | |
| Paridae (Chickadees and Titmice) | 566 | 306 | 248 | 199 | 1319 | 17.69 |  |  |
| Icteridae (Blackbirds) | 341 | 65 | 223 | 93 | 722 | 9.68 |  |  |
| Turdidae (Thrushes) | 199 | 161 | 73 | 226 | 659 | 8.84 |  |  |
| Parulidae (Wood-Warblers) | 306 | 47 | 152 | 96 | 601 | 8.06 |  |  |
| Emberizidae (New World Sparrow) | 277 | 107 | 24 | 89 | 497 | 6.67 |  |  |
| Corvidae (Jays and Crows) | 206 | 105 | 100 | 83 | 494 | 6.63 |  |  |
| Fringillidae (Finches and Allies) | 169 | 105 | 31 | 51 | 356 | 4.77 |  |  |
| Cardinalidae (Cardinals and Tanagers) | 63 | 97 | 51 | 85 | 296 | 3.97 |  |  |
| Sittidae (Nuthatches) | 119 | 67 | 57 | 15 | 258 | 3.46 |  |  |
| Mimidae (Mockingbirds and Thrashers) | 62 | 73 | 27 | 64 | 226 | 3.03 |  |  |
| Hirundinidae (Swallows) | 146 | 7 | 6 | 50 | 209 | 2.80 |  |  |
| Tyrannidae (Tyrant Flycatchers) | 107 | 43 | 30 | 16 | 196 | 2.63 |  |  |
| Vireonidae (Vireos) | 59 | 25 | 45 | 14 | 143 | 1.92 |  |  |
| Passeridae (Old World Sparrow) |  | 52 |  | 67 | 119 | 1.60 |  |  |
| Sturnidae (Starlings) | 5 |  | 2 | 83 | 90 | 1.21 |  |  |
| Troglodytidae (Wrens) | 16 | 21 | 4 | 39 | 80 | 1.07 |  |  |
| Bombycillidae (Waxwings) | 14 | 8 |  | 53 | 75 | 1.01 |  |  |
| Regulidae (Kinglets) | 30 | 8 | 12 |  | 50 | 0.67 |  |  |
| Polioptilidae (Gnatcatchers) | 10 | 11 | 4 | 2 | 27 | 0.36 |  |  |
| Certhiidae (Creepers) | 8 |  | 2 |  | 10 | 0.13 |  |  |
| **Piciformes** |  |  |  |  |  |  |  |  |
| Picidae (Woodpeckers) | 147 | 130 | 72 | 38 | 387 | 5.19 |  |  |
| **Anseriformes** |  |  |  |  |  |  |  |  |
| Anatidae (Ducks, Geese, and Swans) | 222 | 4 | 5 | 11 | 242 | 3.25 |  |  |
| **Columbiformes** |  |  |  |  |  |  |  |  |
| Columbidae (Pigeons and Doves) | 65 | 25 | 7 | 24 | 121 | 1.62 |  |  |
| **Accipitriformes** |  |  |  |  |  |  |  |  |
| Accipitridae (Hawks and Eagles) | 29 | 24 | 23 | 6 | 82 | 1.10 |  |  |
| Cathartidae (Vultures) | 6 | 8 | 6 | 4 | 24 | 0.32 |  |  |
| Pandionidae (Ospreys) | 2 |  |  | 2 | 4 | 0.05 |  |  |
| **Suliformes** |  |  |  |  |  |  |  |  |
| Phalacrocoracidae (Cormorants) | 2 |  |  | 50 | 52 | 0.70 |  |  |
| **Pelecaniformes** |  |  |  |  |  |  |  |  |
| Ardeidae (Herons, Bitterns, and Allies) | 26 | 1 |  | 3 | 30 | 0.40 |  |  |
| **Apodiformes** |  |  |  |  |  |  |  |  |
| Trochilidae (Hummingbirds) | 7 | 1 | 2 | 15 | 25 | 0.34 |  |  |
| Apodidae (Swifts) | 2 | 5 |  | 1 | 8 | 0.11 |  |  |
| **Galliformes** |  |  |  |  |  |  |  |  |
| Phasianidae (Turkeys, Grouse, and Quail) | 10 | 3 |  | 9 | 22 | 0.30 |  |  |
| **Cuculiformes** |  |  |  |  |  |  |  |  |
| Cuculidae (Cuckoos) | 9 | 2 | 2 | 4 | 17 | 0.23 |  |  |
| **Charadriiformes** |  |  |  |  |  |  |  |  |
| Laridae (Gulls, Terns, and Skimmers) | 1 |  |  | 4 | 5 | 0.07 |  |  |
| Scolopacidae (Sandpipers and Allies) | 3 |  |  |  | 3 | 0.04 |  |  |
| **Strigiformes** |  |  |  |  |  |  |  |  |
| Strigidae (Typical Owls) | 3 |  |  | 1 | 4 | 0.05 |  |  |
| **Coraciiformes** |  |  |  |  |  |  |  |  |
| Alcedinidae (Kingfishers) |  |  |  | 2 | 2 | 0.03 |  |  |
| **Falconiformes** |  |  |  |  |  |  |  |  |
| Falconidae (Falcons) | 1 |  |  |  | 1 | 0.01 |  |  |
| Total | 3238 | 1511 | 1208 | 1499 | 7456 |  |  |  |

**Table H.** Frequencies of 99 avian species (in descending order from most to least frequently observed) based on point count data in Chester, CT, April through October, 2010 – 2011 (No. of sites = 7, No. of site visits = 43, comprising 301 point counts.)

| **Avian (common name)** | **Scientific Name** | **April** | **May** | **June** | **July** | **August** | **Sept** | **Oct** | **Avg. Frequency** |
| --- | --- | --- | --- | --- | --- | --- | --- | --- | --- |
| Tufted Titmouse | *Baeolophus bicolor* | 1.000 | 1.000 | 1.000 | 1.000 | 1.000 | 1.000 | 1.000 | 1.000 |
| Black-capped Chickadee | *Poecile atricapillus* | 1.000 | 1.000 | 0.857 | 1.000 | 1.000 | 0.833 | 1.000 | 0.953 |
| American Goldfinch | *Spinus tristis* | 0.833 | 0.833 | 1.000 | 1.000 | 1.000 | 0.833 | 0.600 | 0.884 |
| American Robin | *Turdus migratorius* | 1.000 | 1.000 | 1.000 | 1.000 | 1.000 | 0.500 | 0.600 | 0.884 |
| Blue Jay | *Cyanocitta cristata* | 1.000 | 0.667 | 0.571 | 1.000 | 1.000 | 1.000 | 1.000 | 0.884 |
| White-breasted Nuthatch | *Sitta carolinensis* | 1.000 | 0.500 | 0.714 | 1.000 | 1.000 | 1.000 | 1.000 | 0.884 |
| Downy Woodpecker | *Picoides pubescens* | 0.667 | 0.167 | 0.571 | 1.000 | 1.000 | 1.000 | 0.800 | 0.744 |
| Eastern Towhee | *Pipilo erythrophthalmus* | 0.333 | 0.833 | 1.000 | 1.000 | 0.667 | 0.500 | 0.200 | 0.674 |
| Northern Cardinal | *Cardinalis cardinalis* | 0.833 | 0.667 | 0.857 | 0.857 | 1.000 | 0.167 | 0.200 | 0.674 |
| Mourning Dove | *Zenaida macroura* | 0.500 | 1.000 | 1.000 | 1.000 | 0.667 | 0.167 | 0.000 | 0.651 |
| Black-and-white Warbler | *Mniotilta varia* | 0.333 | 1.000 | 0.857 | 0.571 | 1.000 | 0.500 | 0.000 | 0.628 |
| Gray Catbird | *Dumetella carolinensis* | 0.000 | 1.000 | 1.000 | 1.000 | 0.833 | 0.333 | 0.000 | 0.628 |
| Red-bellied Woodpecker | *Melanerpes carolinus* | 0.500 | 0.833 | 0.286 | 0.429 | 0.667 | 1.000 | 0.800 | 0.628 |
| Eastern Phoebe | *Sayornis phoebe* | 0.500 | 0.167 | 0.571 | 0.857 | 0.667 | 0.333 | 0.800 | 0.558 |
| Eastern Wood-Pewee | *Contopus virens* | 0.000 | 0.333 | 1.000 | 0.857 | 1.000 | 0.500 | 0.000 | 0.558 |
| Barn Swallow | *Hirundo rustica* | 0.167 | 1.000 | 1.000 | 0.857 | 0.500 | 0.000 | 0.000 | 0.535 |
| Chipping Sparrow | *Spizella passerina* | 0.667 | 0.333 | 0.857 | 0.857 | 0.167 | 0.500 | 0.200 | 0.535 |
| Black-throated Green Warbler | *Setophaga virens* | 0.000 | 1.000 | 0.857 | 0.857 | 0.500 | 0.167 | 0.000 | 0.512 |
| Common Grackle | *Quiscalus quiscula* | 0.667 | 0.667 | 0.714 | 0.429 | 0.667 | 0.000 | 0.400 | 0.512 |
| Red-winged Blackbird | *Agelaius phoeniceus* | 1.000 | 0.500 | 1.000 | 0.714 | 0.167 | 0.000 | 0.000 | 0.512 |
| Brown-headed Cowbird | *Molothrus ater* | 0.833 | 0.667 | 0.857 | 0.571 | 0.000 | 0.000 | 0.200 | 0.465 |
| Eastern Kingbird | *Tyrannus tyrannus* | 0.000 | 0.667 | 0.714 | 0.857 | 0.500 | 0.000 | 0.000 | 0.419 |
| Hooded Warbler | *Setophaga citrina* | 0.000 | 0.833 | 1.000 | 0.429 | 0.500 | 0.000 | 0.000 | 0.419 |
| Tree Swallow | *Tachycineta bicolor* | 0.833 | 0.833 | 0.429 | 0.286 | 0.500 | 0.000 | 0.000 | 0.419 |
| American Crow | *Corvus brachyrhynchos* | 0.167 | 0.167 | 0.429 | 0.286 | 0.500 | 0.667 | 0.600 | 0.395 |
| Canada Goose | *Branta canadensis* | 0.500 | 0.167 | 0.714 | 0.571 | 0.000 | 0.167 | 0.600 | 0.395 |
| Wood Duck | *Aix sponsa* | 0.500 | 0.667 | 0.429 | 0.429 | 0.333 | 0.000 | 0.400 | 0.395 |
| Northern Flicker | *Colaptes auratus* | 0.500 | 0.333 | 0.429 | 0.571 | 0.000 | 0.333 | 0.400 | 0.372 |
| Great Blue Heron | *Ardea herodias* | 0.667 | 0.333 | 0.571 | 0.429 | 0.000 | 0.333 | 0.000 | 0.349 |
| Ovenbird | *Seiurus aurocapilla* | 0.000 | 1.000 | 1.000 | 0.286 | 0.000 | 0.000 | 0.000 | 0.349 |
| Warbling Vireo | *Vireo gilvus* | 0.000 | 1.000 | 1.000 | 0.286 | 0.000 | 0.000 | 0.000 | 0.349 |
| Baltimore Oriole | *Icterus galbula* | 0.000 | 1.000 | 0.714 | 0.143 | 0.167 | 0.167 | 0.000 | 0.326 |
| Mallard | *Anas platyrhynchos* | 0.167 | 0.333 | 0.286 | 0.286 | 0.167 | 0.500 | 0.600 | 0.326 |
| Northern Waterthrush | *Parkesia noveboracensis* | 0.333 | 1.000 | 0.857 | 0.000 | 0.000 | 0.000 | 0.000 | 0.326 |
| Red-eyed Vireo | *Vireo olivaceus* | 0.000 | 0.000 | 0.857 | 0.429 | 0.333 | 0.333 | 0.200 | 0.326 |
| Red-shouldered Hawk | *Buteo lineatus* | 0.333 | 0.333 | 0.429 | 0.000 | 0.500 | 0.500 | 0.200 | 0.326 |
| Great Crested Flycatcher | *Myiarchus crinitus* | 0.000 | 0.500 | 0.857 | 0.143 | 0.500 | 0.000 | 0.000 | 0.302 |
| Pine Warbler | *Setophaga pinus* | 0.667 | 0.667 | 0.143 | 0.571 | 0.000 | 0.000 | 0.000 | 0.302 |
| Pileated Woodpecker | *Dryocopus pileatus* | 0.667 | 0.333 | 0.286 | 0.286 | 0.000 | 0.167 | 0.200 | 0.279 |
| Song Sparrow | *Melospiza melodia* | 0.000 | 0.167 | 0.429 | 0.286 | 0.333 | 0.167 | 0.600 | 0.279 |
| Scarlet Tanager | *Piranga olivacea* | 0.000 | 0.333 | 0.857 | 0.286 | 0.167 | 0.000 | 0.000 | 0.256 |
| Eastern Bluebird | *Sialia sialis* | 0.333 | 0.167 | 0.286 | 0.000 | 0.000 | 0.333 | 0.600 | 0.233 |
| Yellow-throated Vireo | *Vireo flavifrons* | 0.000 | 0.667 | 0.286 | 0.429 | 0.000 | 0.167 | 0.000 | 0.233 |
| Carolina Wren | *Thryothorus ludovicianus* | 0.167 | 0.167 | 0.000 | 0.429 | 0.167 | 0.167 | 0.400 | 0.209 |
| Hairy Woodpecker | *Picoides villosus* | 0.333 | 0.167 | 0.429 | 0.286 | 0.000 | 0.000 | 0.200 | 0.209 |
| Louisiana Waterthrush | *Parkesia motacilla* | 0.333 | 0.667 | 0.429 | 0.000 | 0.000 | 0.000 | 0.000 | 0.209 |
| Wood Thrush | *Hylocichla mustelina* | 0.000 | 0.333 | 0.429 | 0.429 | 0.000 | 0.167 | 0.000 | 0.209 |
| Worm-eating Warbler | *Helmitheros vermivorum* | 0.000 | 0.333 | 0.571 | 0.286 | 0.167 | 0.000 | 0.000 | 0.209 |
| Blue-gray Gnatcatcher | *Polioptila caerulea* | 0.167 | 0.833 | 0.286 | 0.000 | 0.000 | 0.000 | 0.000 | 0.186 |
| Dark-eyed Junco | *Junco hyemalis* | 0.167 | 0.000 | 0.000 | 0.000 | 0.000 | 0.333 | 1.000 | 0.186 |
| Yellow-rumped Warbler | *Setophaga coronata* | 0.667 | 0.167 | 0.000 | 0.000 | 0.000 | 0.000 | 0.600 | 0.186 |
| American Redstart | *Setophaga ruticilla* | 0.000 | 0.167 | 0.000 | 0.000 | 0.500 | 0.500 | 0.000 | 0.163 |
| Brown Creeper | *Certhia americana* | 0.333 | 0.167 | 0.143 | 0.286 | 0.167 | 0.000 | 0.000 | 0.163 |
| Cedar Waxwing | *Bombycilla cedrorum* | 0.000 | 0.667 | 0.429 | 0.000 | 0.000 | 0.000 | 0.000 | 0.163 |
| Golden-crowned Kinglet | *Regulus satrapa* | 0.000 | 0.000 | 0.000 | 0.000 | 0.000 | 0.333 | 1.000 | 0.163 |
| Red-tailed Hawk | *Buteo jamaicensis* | 0.333 | 0.333 | 0.000 | 0.000 | 0.000 | 0.167 | 0.400 | 0.163 |
| Ruby-throated Hummingbird | *Archilochus colubris* | 0.000 | 0.000 | 0.143 | 0.286 | 0.333 | 0.333 | 0.000 | 0.163 |
| White-throated Sparrow | *Zonotrichia albicollis* | 0.333 | 0.167 | 0.000 | 0.000 | 0.000 | 0.000 | 0.800 | 0.163 |
| Yellow-billed Cuckoo | *Coccyzus americanus* | 0.000 | 0.333 | 0.286 | 0.286 | 0.000 | 0.167 | 0.000 | 0.163 |
| Turkey Vulture | *Cathartes aura* | 0.167 | 0.000 | 0.286 | 0.000 | 0.000 | 0.167 | 0.200 | 0.116 |
| Veery | *Catharus fuscescens* | 0.000 | 0.500 | 0.143 | 0.000 | 0.167 | 0.000 | 0.000 | 0.116 |
| European Starling | *Sturnus vulgaris* | 0.333 | 0.167 | 0.000 | 0.000 | 0.000 | 0.000 | 0.200 | 0.093 |
| Ruby-crowned Kinglet | *Regulus calendula* | 0.167 | 0.000 | 0.000 | 0.000 | 0.000 | 0.167 | 0.400 | 0.093 |
| Blue-headed Vireo | *Vireo solitarius* | 0.000 | 0.000 | 0.000 | 0.000 | 0.000 | 0.000 | 0.600 | 0.070 |
| House Wren | *Troglodytes aedon* | 0.000 | 0.000 | 0.000 | 0.143 | 0.333 | 0.000 | 0.000 | 0.070 |
| Rose-breasted Grosbeak | *Pheucticus ludovicianus* | 0.000 | 0.167 | 0.143 | 0.143 | 0.000 | 0.000 | 0.000 | 0.070 |
| Black-billed Cuckoo | *Coccyzus erythropthalmus* | 0.000 | 0.000 | 0.143 | 0.143 | 0.000 | 0.000 | 0.000 | 0.047 |
| Black-throated Blue Warbler | *Setophaga caerulescens* | 0.000 | 0.167 | 0.000 | 0.000 | 0.000 | 0.000 | 0.200 | 0.047 |
| Chimney Swift | *Chaetura pelagica* | 0.000 | 0.000 | 0.000 | 0.143 | 0.167 | 0.000 | 0.000 | 0.047 |
| Double-crested Cormorant | *Phalacrocorax auritus* | 0.000 | 0.000 | 0.000 | 0.000 | 0.167 | 0.167 | 0.000 | 0.047 |
| House Finch | *Haemorhous mexicanus* | 0.000 | 0.000 | 0.000 | 0.143 | 0.000 | 0.000 | 0.200 | 0.047 |
| Magnolia Warbler | *Setophaga magnolia* | 0.000 | 0.000 | 0.000 | 0.000 | 0.000 | 0.167 | 0.200 | 0.047 |
| Nashville Warbler | *Oreothlypis ruficapilla* | 0.000 | 0.000 | 0.000 | 0.000 | 0.333 | 0.000 | 0.000 | 0.047 |
| Northern Parula | *Setophaga americana* | 0.000 | 0.167 | 0.000 | 0.000 | 0.000 | 0.167 | 0.000 | 0.047 |
| Osprey | *Pandion haliaetus* | 0.000 | 0.167 | 0.000 | 0.000 | 0.000 | 0.000 | 0.200 | 0.047 |
| Palm Warbler | *Setophaga palmarum* | 0.167 | 0.000 | 0.000 | 0.000 | 0.000 | 0.000 | 0.200 | 0.047 |
| Ring-necked Duck | *Aythya collaris* | 0.333 | 0.000 | 0.000 | 0.000 | 0.000 | 0.000 | 0.000 | 0.047 |
| Sharp-shinned Hawk | *Accipiter striatus* | 0.000 | 0.000 | 0.000 | 0.000 | 0.167 | 0.000 | 0.200 | 0.047 |
| Wild Turkey | *Meleagris gallopavo* | 0.167 | 0.000 | 0.000 | 0.000 | 0.167 | 0.000 | 0.000 | 0.047 |
| Yellow Warbler | *Setophaga petechia* | 0.000 | 0.333 | 0.000 | 0.000 | 0.000 | 0.000 | 0.000 | 0.047 |
| Yellow-bellied Sapsucker | *Sphyrapicus varius* | 0.000 | 0.000 | 0.000 | 0.000 | 0.000 | 0.000 | 0.400 | 0.047 |
| American Black Duck | *Anas rubripes* | 0.167 | 0.000 | 0.000 | 0.000 | 0.000 | 0.000 | 0.000 | 0.023 |
| Barred Owl | *Strix varia* | 0.000 | 0.000 | 0.143 | 0.000 | 0.000 | 0.000 | 0.000 | 0.023 |
| Blackburnian Warbler | *Setophaga fusca* | 0.000 | 0.167 | 0.000 | 0.000 | 0.000 | 0.000 | 0.000 | 0.023 |
| Chestnut-sided Warbler | *Setophaga pensylvanica* | 0.000 | 0.000 | 0.000 | 0.000 | 0.000 | 0.167 | 0.000 | 0.023 |
| Chicken | *Gallus gallus domesticus* | 0.000 | 0.000 | 0.000 | 0.000 | 0.000 | 0.167 | 0.000 | 0.023 |
| Common Raven | *Corvus corax* | 0.000 | 0.000 | 0.000 | 0.000 | 0.000 | 0.000 | 0.200 | 0.023 |
| Common Yellowthroat | *Geothlypis trichas* | 0.000 | 0.000 | 0.000 | 0.000 | 0.000 | 0.000 | 0.200 | 0.023 |
| Cooper's Hawk | *Accipiter cooperii* | 0.000 | 0.000 | 0.000 | 0.000 | 0.167 | 0.000 | 0.000 | 0.023 |
| Fish Crow | *Corvus ossifragus* | 0.000 | 0.167 | 0.000 | 0.000 | 0.000 | 0.000 | 0.000 | 0.023 |
| Great Horned Owl | *Bubo virginianus* | 0.000 | 0.000 | 0.000 | 0.000 | 0.000 | 0.167 | 0.000 | 0.023 |
| Hermit Thrush | *Catharus guttatus* | 0.000 | 0.000 | 0.000 | 0.000 | 0.000 | 0.000 | 0.200 | 0.023 |
| Herring Gull | *Larus argentatus* | 0.167 | 0.000 | 0.000 | 0.000 | 0.000 | 0.000 | 0.000 | 0.023 |
| Merlin | *Falco columbarius* | 0.000 | 0.000 | 0.000 | 0.000 | 0.000 | 0.000 | 0.200 | 0.023 |
| Orchard Oriole | *Icterus spurius* | 0.000 | 0.167 | 0.000 | 0.000 | 0.000 | 0.000 | 0.000 | 0.023 |
| Prairie Warbler | *Setophaga discolor* | 0.000 | 0.000 | 0.143 | 0.000 | 0.000 | 0.000 | 0.000 | 0.023 |
| Red-breasted Nuthatch | *Sitta canadensis* | 0.000 | 0.000 | 0.000 | 0.000 | 0.000 | 0.000 | 0.200 | 0.023 |
| Rusty Blackbird | *Euphagus carolinus* | 0.000 | 0.000 | 0.000 | 0.000 | 0.000 | 0.000 | 0.200 | 0.023 |
| Solitary Sandpiper | *Tringa solitaria* | 0.000 | 0.000 | 0.000 | 0.000 | 0.000 | 0.167 | 0.000 | 0.023 |

Cells highlighted in gray indicate bird species that served as the source of blood meals for *Culiseta melanura*.

**Table I.** Frequencies of 66 avian species (in descending order from most to least

frequently observed) based on point count data in Killingworth, CT, April

through October, 2010 – 2011 (No. of sites = 3, No of site visits = 44, comprising 132

point counts.)

| **Avian (common name)** | **Scientific Name** | **April** | **May** | **June** | **July** | **August** | **Sept** | **Oct** | **Avg. Frequency** |
| --- | --- | --- | --- | --- | --- | --- | --- | --- | --- |
| Tufted Titmouse | *Baeolophus bicolor* | 1.000 | 1.000 | 1.000 | 1.000 | 1.000 | 1.000 | 1.000 | 1.000 |
| Northern Cardinal | *Cardinalis cardinalis* | 1.000 | 1.000 | 1.000 | 1.000 | 1.000 | 1.000 | 0.250 | 0.932 |
| American Robin | *Turdus migratorius* | 1.000 | 1.000 | 1.000 | 1.000 | 0.750 | 0.500 | 0.750 | 0.864 |
| Red-bellied Woodpecker | *Melanerpes carolinus* | 1.000 | 1.000 | 0.750 | 1.000 | 0.500 | 1.000 | 1.000 | 0.864 |
| Black-capped Chickadee | *Poecile atricapillus* | 0.833 | 0.857 | 0.625 | 0.800 | 1.000 | 1.000 | 0.750 | 0.841 |
| Gray Catbird | *Dumetella carolinensis* | 0.000 | 1.000 | 1.000 | 1.000 | 0.875 | 1.000 | 0.250 | 0.773 |
| American Goldfinch | *Spinus tristis* | 0.833 | 0.857 | 0.875 | 1.000 | 0.875 | 0.333 | 0.250 | 0.750 |
| Downy Woodpecker | *Picoides pubescens* | 0.333 | 0.714 | 0.875 | 0.800 | 0.750 | 1.000 | 0.750 | 0.750 |
| Blue Jay | *Cyanocitta cristata* | 0.667 | 0.429 | 0.500 | 0.600 | 0.875 | 1.000 | 1.000 | 0.705 |
| White-breasted Nuthatch | *Sitta carolinensis* | 0.167 | 0.714 | 0.500 | 0.800 | 0.875 | 1.000 | 0.750 | 0.682 |
| Chipping Sparrow | *Spizella passerina* | 0.667 | 1.000 | 1.000 | 0.800 | 0.500 | 0.167 | 0.000 | 0.636 |
| Brown-headed Cowbird | *Molothrus ater* | 0.833 | 0.714 | 0.875 | 0.600 | 0.125 | 0.000 | 0.000 | 0.477 |
| Carolina Wren | *Thryothorus ludovicianus* | 0.333 | 0.714 | 0.375 | 0.600 | 0.500 | 0.167 | 0.500 | 0.455 |
| Mourning Dove | *Zenaida macroura* | 0.500 | 0.571 | 0.750 | 0.600 | 0.125 | 0.500 | 0.000 | 0.455 |
| House Sparrow | *Passer domesticus* | 0.000 | 0.286 | 0.750 | 1.000 | 0.375 | 0.167 | 0.000 | 0.386 |
| Red-shouldered Hawk | *Buteo lineatus* | 0.167 | 0.286 | 0.125 | 0.200 | 0.625 | 0.667 | 0.750 | 0.386 |
| House Finch | *Haemorhous mexicanus* | 1.000 | 0.714 | 0.375 | 0.200 | 0.000 | 0.000 | 0.000 | 0.341 |
| American Crow | *Corvus brachyrhynchos* | 0.000 | 0.571 | 0.000 | 0.800 | 0.500 | 0.000 | 0.500 | 0.318 |
| Red-eyed Vireo | *Vireo olivaceus* | 0.000 | 0.429 | 0.875 | 0.000 | 0.125 | 0.500 | 0.000 | 0.318 |
| Great Crested Flycatcher | *Myiarchus crinitus* | 0.000 | 0.857 | 0.500 | 0.400 | 0.125 | 0.000 | 0.000 | 0.296 |
| Common Grackle | *Quiscalus quiscula* | 0.000 | 0.143 | 0.375 | 0.200 | 0.625 | 0.333 | 0.000 | 0.273 |
| Northern Flicker | *Colaptes auratus* | 0.333 | 0.143 | 0.500 | 0.600 | 0.125 | 0.167 | 0.000 | 0.273 |
| Eastern Phoebe | *Sayornis phoebe* | 0.500 | 0.571 | 0.375 | 0.000 | 0.125 | 0.000 | 0.000 | 0.250 |
| Eastern Wood-Pewee | *Contopus virens* | 0.000 | 0.000 | 0.375 | 0.800 | 0.250 | 0.333 | 0.000 | 0.250 |
| Wood Thrush | *Hylocichla mustelina* | 0.000 | 0.429 | 0.625 | 0.000 | 0.125 | 0.167 | 0.000 | 0.227 |
| Blue-gray Gnatcatcher | *Polioptila caerulea* | 0.000 | 0.429 | 0.500 | 0.400 | 0.000 | 0.000 | 0.000 | 0.205 |
| Northern Waterthrush | *Parkesia noveboracensis* | 0.000 | 0.714 | 0.125 | 0.000 | 0.000 | 0.000 | 0.000 | 0.136 |
| Tree Swallow | *Tachycineta bicolor* | 0.000 | 0.000 | 0.125 | 0.400 | 0.250 | 0.167 | 0.000 | 0.136 |
| Veery | *Catharus fuscescens* | 0.000 | 0.143 | 0.500 | 0.200 | 0.000 | 0.000 | 0.000 | 0.136 |
| Black-and-white Warbler | *Mniotilta varia* | 0.000 | 0.286 | 0.000 | 0.000 | 0.125 | 0.333 | 0.000 | 0.114 |
| Fish Crow | *Corvus ossifragus* | 0.500 | 0.000 | 0.125 | 0.000 | 0.000 | 0.167 | 0.000 | 0.114 |
| Northern Parula | *Setophaga americana* | 0.000 | 0.571 | 0.000 | 0.000 | 0.000 | 0.167 | 0.000 | 0.114 |
| Turkey Vulture | *Cathartes aura* | 0.167 | 0.286 | 0.125 | 0.000 | 0.000 | 0.167 | 0.000 | 0.114 |
| White-throated Sparrow | *Zonotrichia albicollis* | 0.333 | 0.000 | 0.000 | 0.000 | 0.000 | 0.167 | 0.500 | 0.114 |
| American Redstart | *Setophaga ruticilla* | 0.000 | 0.143 | 0.000 | 0.000 | 0.250 | 0.167 | 0.000 | 0.091 |
| Baltimore Oriole | *Icterus galbula* | 0.000 | 0.429 | 0.000 | 0.000 | 0.125 | 0.000 | 0.000 | 0.091 |
| Common Yellowthroat | *Geothlypis trichas* | 0.000 | 0.429 | 0.125 | 0.000 | 0.000 | 0.000 | 0.000 | 0.091 |
| Ovenbird | *Seiurus aurocapilla* | 0.000 | 0.143 | 0.125 | 0.000 | 0.000 | 0.333 | 0.000 | 0.091 |
| Black-throated Green Warbler | *Setophaga virens* | 0.000 | 0.286 | 0.000 | 0.000 | 0.125 | 0.000 | 0.000 | 0.068 |
| Blue-headed Vireo | *Vireo solitarius* | 0.000 | 0.000 | 0.000 | 0.000 | 0.000 | 0.333 | 0.250 | 0.068 |
| Blue-winged Warbler | *Vermivora cyanoptera* | 0.000 | 0.143 | 0.125 | 0.000 | 0.000 | 0.167 | 0.000 | 0.068 |
| Cedar Waxwing | *Bombycilla cedrorum* | 0.000 | 0.000 | 0.375 | 0.000 | 0.000 | 0.000 | 0.000 | 0.068 |
| Chicken | *Gallus gallus domesticus* | 0.167 | 0.000 | 0.000 | 0.000 | 0.000 | 0.000 | 0.500 | 0.068 |
| Chimney Swift | *Chaetura pelagica* | 0.000 | 0.143 | 0.250 | 0.000 | 0.000 | 0.000 | 0.000 | 0.068 |
| Pine Warbler | *Setophaga pinus* | 0.167 | 0.000 | 0.125 | 0.000 | 0.000 | 0.167 | 0.000 | 0.068 |
| Scarlet Tanager | *Piranga olivacea* | 0.000 | 0.286 | 0.125 | 0.000 | 0.000 | 0.000 | 0.000 | 0.068 |
| Black-throated Blue Warbler | *Setophaga caerulescens* | 0.000 | 0.286 | 0.000 | 0.000 | 0.000 | 0.000 | 0.000 | 0.046 |
| Canada Goose | *Branta canadensis* | 0.333 | 0.000 | 0.000 | 0.000 | 0.000 | 0.000 | 0.000 | 0.046 |
| Cooper's Hawk | *Accipiter cooperii* | 0.000 | 0.000 | 0.000 | 0.200 | 0.000 | 0.167 | 0.000 | 0.046 |
| Golden-crowned Kinglet | *Regulus satrapa* | 0.000 | 0.000 | 0.000 | 0.000 | 0.000 | 0.000 | 0.500 | 0.046 |
| Pileated Woodpecker | *Dryocopus pileatus* | 0.000 | 0.000 | 0.000 | 0.000 | 0.125 | 0.167 | 0.000 | 0.046 |
| Red-tailed Hawk | *Buteo jamaicensis* | 0.000 | 0.143 | 0.000 | 0.000 | 0.125 | 0.000 | 0.000 | 0.046 |
| Dark-eyed Junco | *Junco hyemalis* | 0.167 | 0.000 | 0.000 | 0.000 | 0.000 | 0.000 | 0.250 | 0.046 |
| Yellow-billed Cuckoo | *Coccyzus americanus* | 0.000 | 0.000 | 0.125 | 0.200 | 0.000 | 0.000 | 0.000 | 0.046 |
| Yellow-rumped Warbler | *Setophaga coronata* | 0.167 | 0.000 | 0.000 | 0.000 | 0.000 | 0.000 | 0.250 | 0.046 |
| Broad-winged Hawk | *Buteo platypterus* | 0.000 | 0.143 | 0.000 | 0.000 | 0.000 | 0.000 | 0.000 | 0.023 |
| Eastern Bluebird | *Sialia sialis* | 0.000 | 0.000 | 0.000 | 0.000 | 0.125 | 0.000 | 0.000 | 0.023 |
| Great Blue Heron | *Ardea herodias* | 0.000 | 0.143 | 0.000 | 0.000 | 0.000 | 0.000 | 0.000 | 0.023 |
| Hairy Woodpecker | *Picoides villosus* | 0.000 | 0.000 | 0.000 | 0.000 | 0.000 | 0.167 | 0.000 | 0.023 |
| Magnolia Warbler | *Setophaga magnolia* | 0.000 | 0.143 | 0.000 | 0.000 | 0.000 | 0.000 | 0.000 | 0.023 |
| Red-breasted Nuthatch | *Sitta canadensis* | 0.000 | 0.000 | 0.000 | 0.000 | 0.000 | 0.167 | 0.000 | 0.023 |
| Rose-breasted Grosbeak | *Pheucticus ludovicianus* | 0.000 | 0.143 | 0.000 | 0.000 | 0.000 | 0.000 | 0.000 | 0.023 |
| Ruby-crowned Kinglet | *Regulus calendula* | 0.000 | 0.000 | 0.000 | 0.000 | 0.000 | 0.000 | 0.250 | 0.023 |
| Ruby-throated Hummingbird | *Archilochus colubris* | 0.000 | 0.000 | 0.125 | 0.000 | 0.000 | 0.000 | 0.000 | 0.023 |
| Worm-eating Warbler | *Helmitheros vermivorum* | 0.000 | 0.000 | 0.125 | 0.000 | 0.000 | 0.000 | 0.000 | 0.023 |
| Yellow-throated Vireo | *Vireo flavifrons* | 0.000 | 0.000 | 0.000 | 0.000 | 0.125 | 0.000 | 0.000 | 0.023 |

Cells highlighted in gray indicate bird species that served as the source of blood meals for *Culiseta melanura*.

**Table J.** Frequencies of 66 avian species (in descending order from most to least frequently observed) based on point count data in Madison, CT, April through October, 2010 – 2011 (No. of sites = 4, No. of site visits = 43, comprising 172 point counts.)

| **Avian (common name)** | **Scientific Name** | **April** | **May** | **June** | **July** | **August** | **Sept** | **Oct** | **Avg. Frequency** |
| --- | --- | --- | --- | --- | --- | --- | --- | --- | --- |
| Tufted Titmouse | *Baeolophus bicolor* | 0.833 | 0.857 | 1.000 | 0.800 | 1.000 | 1.000 | 1.000 | 0.930 |
| Black-capped Chickadee | *Poecile atricapillus* | 1.000 | 0.571 | 0.625 | 0.800 | 0.857 | 0.833 | 1.000 | 0.791 |
| White-breasted Nuthatch | *Sitta carolinensis* | 0.500 | 0.286 | 0.625 | 0.800 | 1.000 | 1.000 | 1.000 | 0.721 |
| Blue Jay | *Cyanocitta cristata* | 0.500 | 0.714 | 0.500 | 0.600 | 0.714 | 1.000 | 1.000 | 0.698 |
| Downy Woodpecker | *Picoides pubescens* | 0.333 | 0.143 | 1.000 | 0.600 | 0.857 | 0.500 | 1.000 | 0.628 |
| American Robin | *Turdus migratorius* | 0.500 | 0.571 | 0.750 | 0.800 | 0.714 | 0.167 | 0.750 | 0.605 |
| Northern Cardinal | *Cardinalis cardinalis* | 0.833 | 0.714 | 0.875 | 0.800 | 0.429 | 0.000 | 0.000 | 0.558 |
| Red-eyed Vireo | *Vireo olivaceus* | 0.000 | 0.857 | 0.875 | 0.800 | 0.714 | 0.333 | 0.000 | 0.558 |
| Gray Catbird | *Dumetella carolinensis* | 0.000 | 0.571 | 0.500 | 0.600 | 0.857 | 0.333 | 0.000 | 0.442 |
| Red-bellied Woodpecker | *Melanerpes carolinus* | 0.500 | 0.000 | 0.000 | 0.400 | 0.429 | 0.833 | 1.000 | 0.395 |
| American Goldfinch | *Spinus tristis* | 0.500 | 0.143 | 0.250 | 0.800 | 0.714 | 0.000 | 0.250 | 0.372 |
| Ovenbird | *Seiurus aurocapilla* | 0.000 | 1.000 | 1.000 | 0.200 | 0.000 | 0.000 | 0.000 | 0.372 |
| Chipping Sparrow | *Spizella passerina* | 0.333 | 0.429 | 0.750 | 0.800 | 0.000 | 0.000 | 0.000 | 0.349 |
| Great Crested Flycatcher | *Myiarchus crinitus* | 0.000 | 0.714 | 0.750 | 0.400 | 0.286 | 0.000 | 0.000 | 0.349 |
| Wood Thrush | *Hylocichla mustelina* | 0.000 | 0.286 | 0.750 | 0.800 | 0.286 | 0.167 | 0.000 | 0.349 |
| Black-and-white Warbler | *Mniotilta varia* | 0.167 | 1.000 | 0.125 | 0.200 | 0.143 | 0.167 | 0.000 | 0.279 |
| Scarlet Tanager | *Piranga olivacea* | 0.000 | 0.429 | 0.625 | 0.400 | 0.143 | 0.000 | 0.000 | 0.256 |
| Worm-eating Warbler | *Helmitheros vermivorum* | 0.000 | 0.714 | 0.625 | 0.200 | 0.000 | 0.000 | 0.000 | 0.256 |
| Black-throated Green Warbler | *Setophaga virens* | 0.000 | 0.429 | 0.500 | 0.000 | 0.143 | 0.167 | 0.250 | 0.233 |
| Blue-winged Warbler | *Vermivora cyanoptera* | 0.000 | 0.714 | 0.375 | 0.200 | 0.143 | 0.000 | 0.000 | 0.233 |
| Red-shouldered Hawk | *Buteo lineatus* | 0.333 | 0.143 | 0.250 | 0.000 | 0.000 | 0.500 | 0.500 | 0.233 |
| Eastern Phoebe | *Sayornis phoebe* | 0.167 | 0.000 | 0.250 | 0.400 | 0.000 | 0.333 | 0.250 | 0.186 |
| American Crow | *Corvus brachyrhynchos* | 0.167 | 0.000 | 0.375 | 0.000 | 0.143 | 0.167 | 0.250 | 0.163 |
| Common Yellowthroat | *Geothlypis trichas* | 0.000 | 0.143 | 0.375 | 0.200 | 0.000 | 0.167 | 0.000 | 0.140 |
| Mourning Dove | *Zenaida macroura* | 0.167 | 0.000 | 0.250 | 0.400 | 0.143 | 0.000 | 0.000 | 0.140 |
| Pileated Woodpecker | *Dryocopus pileatus* | 0.000 | 0.143 | 0.250 | 0.000 | 0.286 | 0.167 | 0.000 | 0.140 |
| Common Grackle | *Quiscalus quiscula* | 0.000 | 0.000 | 0.000 | 0.400 | 0.143 | 0.333 | 0.000 | 0.116 |
| Eastern Towhee | *Pipilo erythrophthalmus* | 0.000 | 0.286 | 0.250 | 0.200 | 0.000 | 0.000 | 0.000 | 0.116 |
| House Finch | *Haemorhous mexicanus* | 0.167 | 0.286 | 0.125 | 0.000 | 0.143 | 0.000 | 0.000 | 0.116 |
| Yellow-rumped Warbler | *Setophaga coronata* | 0.000 | 0.143 | 0.000 | 0.000 | 0.000 | 0.000 | 1.000 | 0.116 |
| Yellow-throated Vireo | *Vireo flavifrons* | 0.000 | 0.286 | 0.250 | 0.000 | 0.143 | 0.000 | 0.000 | 0.116 |
| Baltimore Oriole | *Icterus galbula* | 0.000 | 0.286 | 0.125 | 0.000 | 0.143 | 0.000 | 0.000 | 0.093 |
| Blue-headed Vireo | *Vireo solitarius* | 0.000 | 0.000 | 0.000 | 0.000 | 0.143 | 0.000 | 0.750 | 0.093 |
| Broad-winged Hawk | *Buteo platypterus* | 0.000 | 0.286 | 0.000 | 0.200 | 0.143 | 0.000 | 0.000 | 0.093 |
| Brown-headed Cowbird | *Molothrus ater* | 0.167 | 0.286 | 0.125 | 0.000 | 0.000 | 0.000 | 0.000 | 0.093 |
| Northern Flicker | *Colaptes auratus* | 0.000 | 0.000 | 0.125 | 0.000 | 0.143 | 0.167 | 0.250 | 0.093 |
| Northern Parula | *Setophaga americana* | 0.000 | 0.429 | 0.000 | 0.000 | 0.143 | 0.000 | 0.000 | 0.093 |
| Northern Waterthrush | *Parkesia noveboracensis* | 0.000 | 0.571 | 0.000 | 0.000 | 0.000 | 0.000 | 0.000 | 0.093 |
| Red-tailed Hawk | *Buteo jamaicensis* | 0.000 | 0.143 | 0.125 | 0.000 | 0.143 | 0.000 | 0.250 | 0.093 |
| Turkey Vulture | *Cathartes aura* | 0.333 | 0.000 | 0.000 | 0.000 | 0.143 | 0.167 | 0.000 | 0.093 |
| American Redstart | *Setophaga ruticilla* | 0.000 | 0.286 | 0.000 | 0.000 | 0.143 | 0.000 | 0.000 | 0.070 |
| Blue-gray Gnatcatcher | *Polioptila caerulea* | 0.167 | 0.000 | 0.250 | 0.000 | 0.000 | 0.000 | 0.000 | 0.070 |
| Carolina Wren | *Thryothorus ludovicianus* | 0.000 | 0.000 | 0.000 | 0.000 | 0.143 | 0.167 | 0.250 | 0.070 |
| Golden-crowned Kinglet | *Regulus satrapa* | 0.000 | 0.000 | 0.000 | 0.000 | 0.000 | 0.000 | 0.750 | 0.070 |
| Ruby-crowned Kinglet | *Regulus calendula* | 0.000 | 0.000 | 0.000 | 0.000 | 0.000 | 0.000 | 0.750 | 0.070 |
| Veery | *Catharus fuscescens* | 0.000 | 0.143 | 0.250 | 0.000 | 0.000 | 0.000 | 0.000 | 0.070 |
| Blackburnian Warbler | *Setophaga fusca* | 0.000 | 0.143 | 0.000 | 0.000 | 0.143 | 0.000 | 0.000 | 0.047 |
| Brown Creeper | *Certhia americana* | 0.167 | 0.000 | 0.000 | 0.000 | 0.000 | 0.000 | 0.250 | 0.047 |
| Canada Goose | *Branta canadensis* | 0.000 | 0.000 | 0.000 | 0.000 | 0.000 | 0.167 | 0.250 | 0.047 |
| Eastern Wood-Pewee | *Contopus virens* | 0.000 | 0.000 | 0.000 | 0.200 | 0.000 | 0.167 | 0.000 | 0.047 |
| Rose-breasted Grosbeak | *Pheucticus ludovicianus* | 0.000 | 0.143 | 0.125 | 0.000 | 0.000 | 0.000 | 0.000 | 0.047 |
| Ruby-throated Hummingbird | *Archilochus colubris* | 0.000 | 0.000 | 0.000 | 0.200 | 0.143 | 0.000 | 0.000 | 0.047 |
| Sharp-shinned Hawk | *Accipiter striatus* | 0.167 | 0.000 | 0.000 | 0.000 | 0.000 | 0.167 | 0.000 | 0.047 |
| Tree Swallow | *Tachycineta bicolor* | 0.000 | 0.000 | 0.000 | 0.200 | 0.143 | 0.000 | 0.000 | 0.047 |
| Yellow-billed Cuckoo | *Coccyzus americanus* | 0.000 | 0.000 | 0.000 | 0.200 | 0.143 | 0.000 | 0.000 | 0.047 |
| Blackpoll Warbler | *Setophaga striata* | 0.000 | 0.143 | 0.000 | 0.000 | 0.000 | 0.000 | 0.000 | 0.023 |
| Black-throated Blue Warbler | *Setophaga caerulescens* | 0.000 | 0.143 | 0.000 | 0.000 | 0.000 | 0.000 | 0.000 | 0.023 |
| Eastern Bluebird | *Sialia sialis* | 0.000 | 0.000 | 0.000 | 0.000 | 0.143 | 0.000 | 0.000 | 0.023 |
| European Starling | *Sturnus vulgaris* | 0.000 | 0.000 | 0.125 | 0.000 | 0.000 | 0.000 | 0.000 | 0.023 |
| Hairy Woodpecker | *Picoides villosus* | 0.000 | 0.000 | 0.000 | 0.000 | 0.143 | 0.000 | 0.000 | 0.023 |
| Hermit Thrush | *Catharus guttatus* | 0.000 | 0.000 | 0.000 | 0.000 | 0.000 | 0.000 | 0.250 | 0.023 |
| Nashville Warbler | *Oreothlypis ruficapilla* | 0.000 | 0.000 | 0.000 | 0.000 | 0.000 | 0.167 | 0.000 | 0.023 |
| Palm Warbler | *Setophaga palmarum* | 0.000 | 0.000 | 0.000 | 0.000 | 0.000 | 0.000 | 0.250 | 0.023 |
| Pine Warbler | *Setophaga pinus* | 0.000 | 0.000 | 0.000 | 0.000 | 0.000 | 0.167 | 0.000 | 0.023 |
| Red-breasted Nuthatch | *Sitta canadensis* | 0.000 | 0.000 | 0.000 | 0.000 | 0.000 | 0.000 | 0.250 | 0.023 |
| Dark-eyed Junco | *Junco hyemalis* | 0.000 | 0.000 | 0.000 | 0.000 | 0.000 | 0.000 | 0.250 | 0.023 |

Cells highlighted in gray indicate bird species that served as the source of blood meals for *Culiseta melanura*.

**Table K.** Frequencies of 68 avian species (in descending order from most to least frequently observed) based on point count data in North Stonington CT, April through October 2011 (No. of sites = 3, No. of site visits = 28, comprising 84 point counts.)

| **Avian (common name)** | **Scientific Name** | **April** | **May** | **June** | **July** | **August** | **Sept** | **Oct** | **Avg. Frequency** |
| --- | --- | --- | --- | --- | --- | --- | --- | --- | --- |
| Black-capped Chickadee | *Poecile atricapillus* | 1.000 | 1.000 | 1.000 | 1.000 | 1.000 | 1.000 | 1.000 | 1.000 |
| Northern Cardinal | *Cardinalis cardinalis* | 1.000 | 1.000 | 1.000 | 1.000 | 1.000 | 0.500 | 0.750 | 0.893 |
| American Robin | *Turdus migratorius* | 0.667 | 1.000 | 1.000 | 0.750 | 1.000 | 0.500 | 1.000 | 0.857 |
| Tufted Titmouse | *Baeolophus bicolor* | 1.000 | 0.750 | 1.000 | 0.750 | 1.000 | 0.750 | 0.750 | 0.857 |
| Gray Catbird | *Dumetella carolinensis* | 0.000 | 1.000 | 1.000 | 1.000 | 1.000 | 1.000 | 0.500 | 0.821 |
| American Goldfinch | *Spinus tristis* | 0.667 | 0.750 | 1.000 | 1.000 | 1.000 | 0.500 | 0.000 | 0.714 |
| House Sparrow | *Passer domesticus* | 0.667 | 0.750 | 1.000 | 0.750 | 0.500 | 0.000 | 1.000 | 0.679 |
| Chipping Sparrow | *Spizella passerina* | 0.667 | 1.000 | 1.000 | 1.000 | 0.500 | 0.000 | 0.250 | 0.643 |
| Blue Jay | *Cyanocitta cristata* | 0.667 | 0.750 | 0.200 | 0.750 | 0.500 | 0.500 | 1.000 | 0.607 |
| House Wren | *Troglodytes aedon* | 0.000 | 1.000 | 1.000 | 1.000 | 0.500 | 0.000 | 0.000 | 0.536 |
| Mourning Dove | *Zenaida macroura* | 0.000 | 0.500 | 0.600 | 1.000 | 1.000 | 0.250 | 0.250 | 0.536 |
| Brown-headed Cowbird | *Molothrus ater* | 1.000 | 1.000 | 0.800 | 0.500 | 0.250 | 0.000 | 0.000 | 0.500 |
| Downy Woodpecker | *Picoides pubescens* | 0.667 | 0.000 | 0.200 | 0.750 | 0.750 | 0.750 | 0.500 | 0.500 |
| Fish Crow | *Corvus ossifragus* | 1.000 | 0.500 | 1.000 | 0.750 | 0.250 | 0.000 | 0.000 | 0.500 |
| White-breasted Nuthatch | *Sitta carolinensis* | 0.667 | 0.000 | 0.200 | 0.750 | 0.500 | 1.000 | 0.500 | 0.500 |
| American Crow | *Corvus brachyrhynchos* | 0.333 | 0.250 | 0.800 | 0.500 | 0.750 | 0.500 | 0.000 | 0.464 |
| Cedar Waxwing | *Bombycilla cedrorum* | 0.000 | 0.000 | 0.800 | 1.000 | 0.750 | 0.000 | 0.500 | 0.464 |
| Eastern Bluebird | *Sialia sialis* | 0.333 | 0.750 | 0.800 | 0.750 | 0.000 | 0.000 | 0.250 | 0.429 |
| Prairie Warbler | *Setophaga discolor* | 0.000 | 1.000 | 1.000 | 0.250 | 0.500 | 0.000 | 0.000 | 0.429 |
| Eastern Phoebe | *Sayornis phoebe* | 0.667 | 0.750 | 0.400 | 0.250 | 0.250 | 0.000 | 0.500 | 0.393 |
| Ruby-throated Hummingbird | *Archilochus colubris* | 0.000 | 0.750 | 0.600 | 0.500 | 0.750 | 0.000 | 0.000 | 0.393 |
| Tree Swallow | *Tachycineta bicolor* | 0.333 | 0.750 | 0.600 | 0.500 | 0.500 | 0.000 | 0.000 | 0.393 |
| Common Grackle | *Quiscalus quiscula* | 0.667 | 0.500 | 0.600 | 0.250 | 0.500 | 0.000 | 0.000 | 0.357 |
| Chicken | *Gallus gallus domesticus* | 1.000 | 0.250 | 0.800 | 0.250 | 0.000 | 0.000 | 0.000 | 0.321 |
| Ovenbird | *Seiurus aurocapilla* | 0.000 | 0.750 | 0.800 | 0.500 | 0.000 | 0.000 | 0.000 | 0.321 |
| Red-eyed Vireo | *Vireo olivaceus* | 0.000 | 0.500 | 1.000 | 0.250 | 0.000 | 0.000 | 0.250 | 0.321 |
| Wood Thrush | *Hylocichla mustelina* | 0.000 | 1.000 | 0.600 | 0.500 | 0.000 | 0.000 | 0.000 | 0.321 |
| Common Yellowthroat | *Geothlypis trichas* | 0.000 | 0.750 | 0.400 | 0.750 | 0.000 | 0.000 | 0.000 | 0.286 |
| European Starling | *Sturnus vulgaris* | 0.000 | 0.750 | 0.200 | 0.250 | 0.000 | 0.250 | 0.500 | 0.286 |
| Northern Flicker | *Colaptes auratus* | 0.667 | 0.000 | 0.000 | 0.250 | 0.250 | 0.500 | 0.500 | 0.286 |
| Barn Swallow | *Hirundo rustica* | 0.000 | 0.000 | 0.600 | 0.250 | 0.500 | 0.000 | 0.000 | 0.214 |
| Northern Waterthrush | *Parkesia noveboracensis* | 0.000 | 0.250 | 0.800 | 0.250 | 0.000 | 0.000 | 0.000 | 0.214 |
| Red-bellied Woodpecker | *Melanerpes carolinus* | 0.333 | 0.250 | 0.000 | 0.000 | 0.250 | 0.000 | 0.750 | 0.214 |
| Blue-winged Warbler | *Vermivora cyanoptera* | 0.000 | 0.750 | 0.000 | 0.500 | 0.000 | 0.000 | 0.000 | 0.179 |
| Indigo Bunting | *Passerina cyanea* | 0.000 | 0.000 | 0.600 | 0.500 | 0.000 | 0.000 | 0.000 | 0.179 |
| Pine Warbler | *Setophaga pinus* | 0.333 | 0.500 | 0.000 | 0.000 | 0.000 | 0.000 | 0.250 | 0.143 |
| Broad-winged Hawk | *Buteo platypterus* | 0.000 | 0.250 | 0.200 | 0.250 | 0.000 | 0.000 | 0.000 | 0.107 |
| Canada Goose | *Branta canadensis* | 0.000 | 0.000 | 0.000 | 0.000 | 0.000 | 0.000 | 0.750 | 0.107 |
| Red-winged Blackbird | *Agelaius phoeniceus* | 0.333 | 0.500 | 0.000 | 0.000 | 0.000 | 0.000 | 0.000 | 0.107 |
| Turkey Vulture | *Cathartes aura* | 0.333 | 0.250 | 0.000 | 0.000 | 0.000 | 0.000 | 0.250 | 0.107 |
| Veery | *Catharus fuscescens* | 0.000 | 0.000 | 0.400 | 0.250 | 0.000 | 0.000 | 0.000 | 0.107 |
| White-throated Sparrow | *Zonotrichia albicollis* | 0.667 | 0.000 | 0.000 | 0.000 | 0.000 | 0.000 | 0.250 | 0.107 |
| Yellow-billed Cuckoo | *Coccyzus americanus* | 0.000 | 0.000 | 0.200 | 0.250 | 0.250 | 0.000 | 0.000 | 0.107 |
| Yellow-rumped Warbler | *Setophaga coronata* | 0.000 | 0.000 | 0.000 | 0.000 | 0.000 | 0.000 | 0.750 | 0.107 |
| American Redstart | *Setophaga ruticilla* | 0.000 | 0.000 | 0.000 | 0.000 | 0.250 | 0.250 | 0.000 | 0.071 |
| Belted Kingfisher | *Megaceryle alcyon* | 0.000 | 0.000 | 0.000 | 0.250 | 0.000 | 0.000 | 0.250 | 0.071 |
| Black-and-white Warbler | *Mniotilta varia* | 0.000 | 0.000 | 0.200 | 0.000 | 0.000 | 0.250 | 0.000 | 0.071 |
| Blue-gray Gnatcatcher | *Polioptila caerulea* | 0.000 | 0.250 | 0.200 | 0.000 | 0.000 | 0.000 | 0.000 | 0.071 |
| Eastern Wood-Pewee | *Contopus virens* | 0.000 | 0.000 | 0.200 | 0.000 | 0.250 | 0.000 | 0.000 | 0.071 |
| Great Blue Heron | *Ardea herodias* | 0.000 | 0.500 | 0.000 | 0.000 | 0.000 | 0.000 | 0.000 | 0.071 |
| Great Crested Flycatcher | *Myiarchus crinitus* | 0.000 | 0.000 | 0.400 | 0.000 | 0.000 | 0.000 | 0.000 | 0.071 |
| Northern Parula | *Setophaga americana* | 0.000 | 0.250 | 0.000 | 0.000 | 0.000 | 0.000 | 0.250 | 0.071 |
| Osprey | *Pandion haliaetus* | 0.333 | 0.000 | 0.200 | 0.000 | 0.000 | 0.000 | 0.000 | 0.071 |
| Pileated Woodpecker | *Dryocopus pileatus* | 0.000 | 0.000 | 0.000 | 0.000 | 0.000 | 0.500 | 0.000 | 0.071 |
| Song Sparrow | *Melospiza melodia* | 0.000 | 0.250 | 0.000 | 0.000 | 0.000 | 0.000 | 0.250 | 0.071 |
| Barred Owl | *Strix varia* | 0.000 | 0.250 | 0.000 | 0.000 | 0.000 | 0.000 | 0.000 | 0.036 |
| Black-billed Cuckoo | *Coccyzus erythropthalmus* | 0.000 | 0.000 | 0.200 | 0.000 | 0.000 | 0.000 | 0.000 | 0.036 |
| Black-throated Blue Warbler | *Setophaga caerulescens* | 0.000 | 0.250 | 0.000 | 0.000 | 0.000 | 0.000 | 0.000 | 0.036 |
| Carolina Wren | *Thryothorus ludovicianus* | 0.000 | 0.000 | 0.000 | 0.000 | 0.000 | 0.000 | 0.250 | 0.036 |
| Chimney Swift | *Chaetura pelagica* | 0.000 | 0.000 | 0.200 | 0.000 | 0.000 | 0.000 | 0.000 | 0.036 |
| Common Raven | *Corvus corax* | 0.000 | 0.000 | 0.000 | 0.000 | 0.000 | 0.250 | 0.000 | 0.036 |
| Cooper's Hawk | *Accipiter cooperii* | 0.000 | 0.000 | 0.000 | 0.000 | 0.000 | 0.000 | 0.250 | 0.036 |
| Double-crested Cormorant | *Phalacrocorax auritus* | 0.000 | 0.000 | 0.000 | 0.000 | 0.250 | 0.000 | 0.000 | 0.036 |
| Eastern Towhee | *Pipilo erythrophthalmus* | 0.000 | 0.000 | 0.000 | 0.000 | 0.000 | 0.000 | 0.250 | 0.036 |
| Herring Gull | *Larus argentatus* | 0.000 | 0.000 | 0.000 | 0.000 | 0.250 | 0.000 | 0.000 | 0.036 |
| Red-shouldered Hawk | *Buteo lineatus* | 0.000 | 0.250 | 0.000 | 0.000 | 0.000 | 0.000 | 0.000 | 0.036 |
| Sharp-shinned Hawk | *Accipiter striatus* | 0.000 | 0.000 | 0.000 | 0.000 | 0.000 | 0.000 | 0.250 | 0.036 |
| Dark-eyed Junco | *Junco hyemalis* | 0.000 | 0.000 | 0.000 | 0.000 | 0.000 | 0.000 | 0.250 | 0.036 |

Cells highlighted in gray indicate bird species that served as the source of blood meals for *Culiseta melanura*.

**Table L.** Feeding index for each host bird species. Feeding index is the relative likelihood of a blood meal on a given bird species per bird of that species, which indicates the preference of mosquitoes for feeding on different bird species. The feeding index for the named bird species are relative to the remaining, other birds, for which the feeding index was chosen to be 1.

| Avian Species | Median Feeding Index | 95% Confidence Interval |
| --- | --- | --- |
| Wood Thrush | 1044.13 | (513.01 , 2300.98) |
| Warbling Vireo | 632.40 | (211.54 , 2116.52) |
| Chipping Sparrow | 39.87 | (24.08 , 66.84) |
| Northern Cardinal | 24.31 | (16.49 , 40.27) |
| American Robin | 14.2 | (10.18 , 20.22) |
| Common Grackle | 7.37 | (5.14 , 10.70) |
| Tufted Titmouse | 6.19 | (4.60 , 8.29) |
| Black-capped Chickadee | 5.11 | (3.35 , 7.63) |
| Other Birds | 1 | — |

A total of 6,234 *Cs. melanura* mosquitoes were collected from the four EEE virus foci in Connecticut, Chester (N = 2,484), Killingworth (N = 1,322), Madison (N = 1,706), and North Stonington (N = 722) by using 120 resting boxes placed at 8 locations. Greater numbers of *Cs. melanura* were collected during 2011 than in 2010 in all four trapping locations. Multiple collection peaks were observed during the trapping season, which suggested 2-3 generations of *Cs. melanura* each year (Fig. S1).
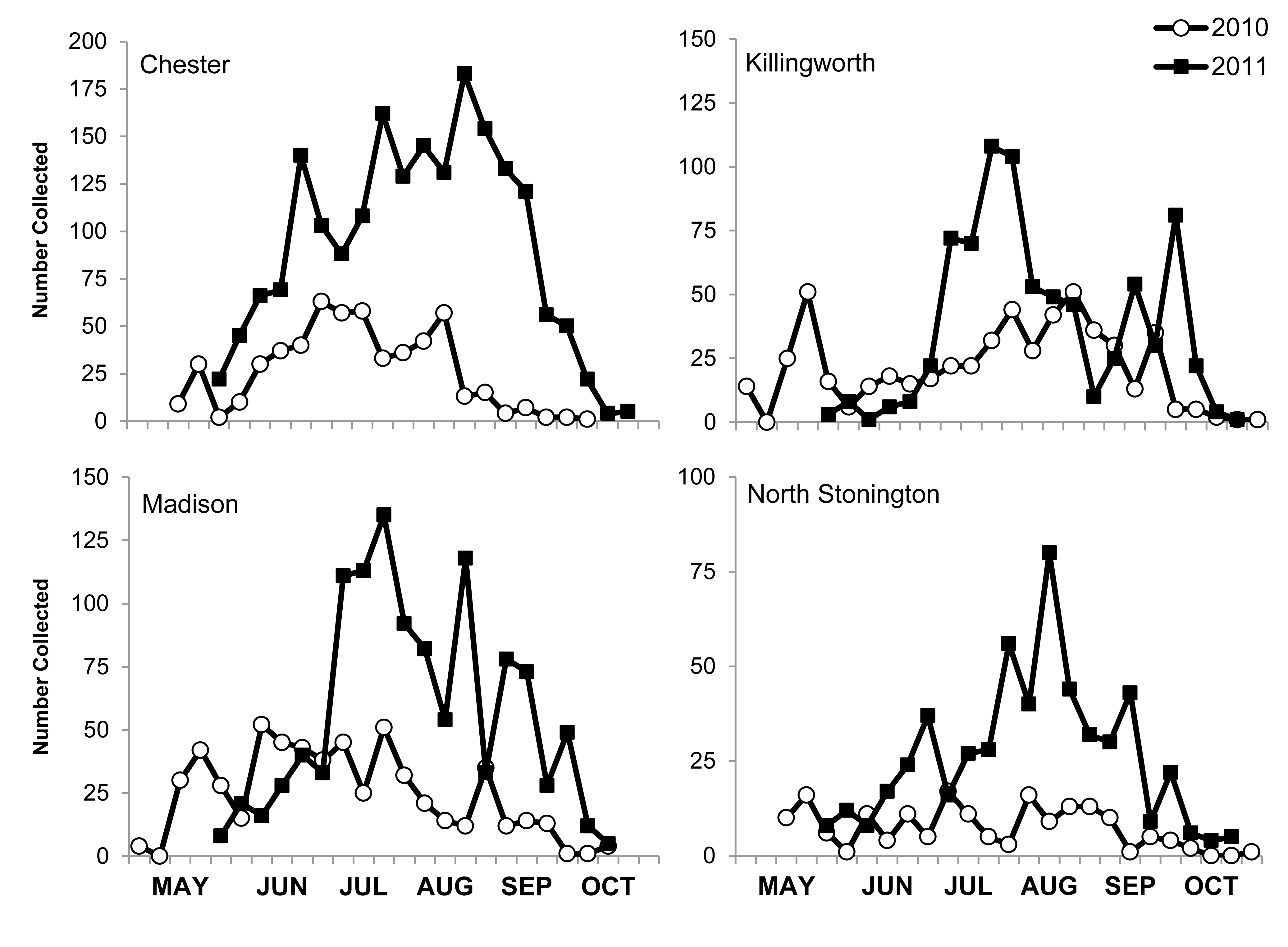


**Fig A.** Population abundance and peak seasonal activity of adult female *Culiseta melanura* in four study sites, Chester, Killingworth, Madison and North Stonington, CT, 2010-2011


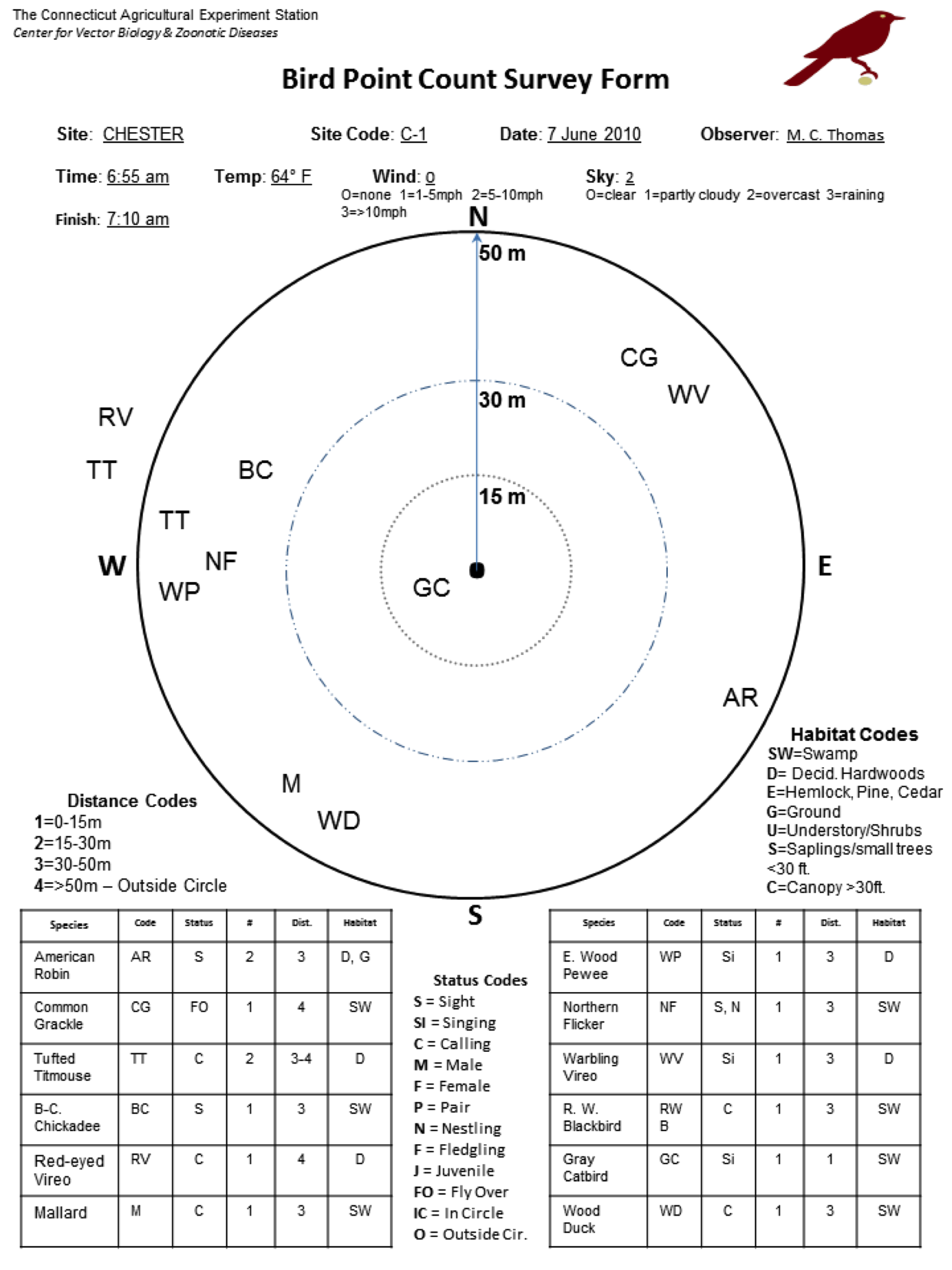


**Fig B. Avian point count surveys form.** Status and habitat codes, including observations of nestlings, fledglings, and juvenile birds, and individuals detected outside or flying over the point count circle.

**Fig C.** **Force of infection due to each host species.** The solid line is the median value over the 1000 samples produced, which the shaded region represents the 95% confidence interval.


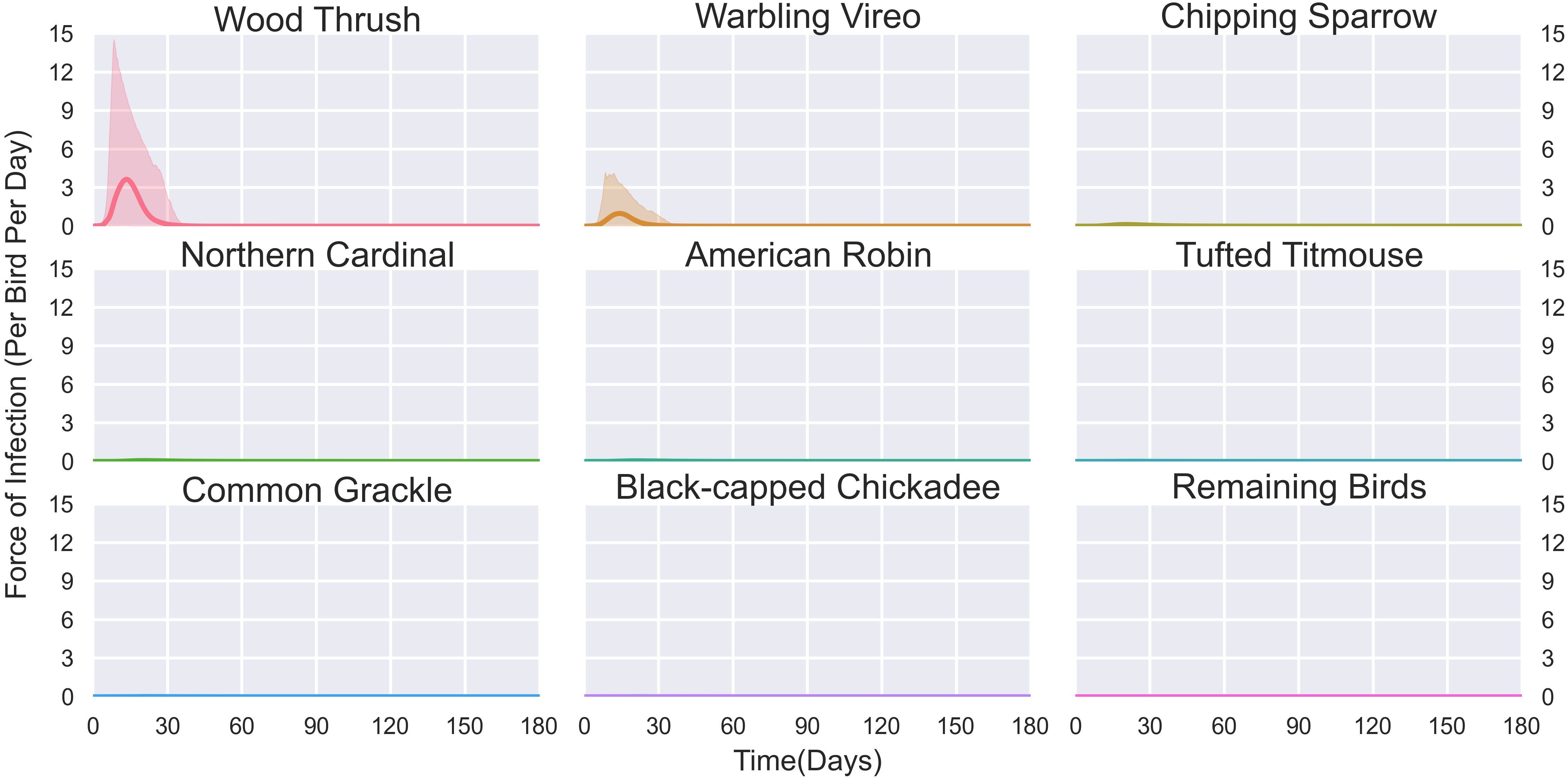


**Modeling Supporting Information**

We choose 8 preferred host species (*i* = 1, 2, ..., 8), and a ninth consisting of all other birds (*i* = 9). To calculate the feeding index $\alpha_{i}$, we note that the proportion of the blood meals that are on bird species is

$$\text{f}_{\text{i}}\text{=}\frac{\text{α}_{\text{i}}\text{N}_{\text{i}}}{\sum_{\text{j=1}}^{\text{n}} \text{α}_{\text{j}}\text{N}_{\text{j}}}\text{}$$

where $N_{i}$ is the number of birds of species *i* in the population. The $\text{f}_{\text{i}}$are simply the proportion of blood meals on the different species from the samples. We assumed the $N_{i}$ were the bird counts, which amounts to assuming that the bird species are equally likely to be observed given that they are present*.* Rearranging the equation gives

$$\text{α}_{\text{i}}\text{N}_{\text{i}}\text{-}\text{f}_{\text{i}}\sum_{\text{j=1}}^{\text{n}} \text{α}_{\text{j}}\text{N}_{\text{j}}\text{=0}$$

which shows that $\alpha$ is an eigenvector of the 0 eigenvalue. We used a standard eigenvalue routine to numerically compute the $\alpha$.

To generate statistics on the feeding index, first the host species were assumed to have arrived via a Poisson process. This assumption gives a gamma likelihood function for the Poisson rate given the data. From this we sampled to get a distribution for the Poisson parameter. Having sampled the parameter value, we sampled from the Poisson processes with these parameter values to get sample bird counts.

The total number of blood meals were assumed to arrive via a Poisson process. This assumption gives a gamma likelihood function for the Poisson rate given the data. From this we sampled to get a distribution for the Poisson parameter. Having sampled the parameter value, we sampled from the Poisson process with these parameter values to get sample total number of blood meals.

The proportion of blood meals on each host species was assumed to arrive via a multinomial process. This gives a likelihood function for the multinomial parameters given the blood meal data. Utilizing the Markov Chain Monte Carlo (MCMC) method with the Metropolis-Hastings algorithm we obtained samples of the multinomial parameters. Having sampled the multinomial parameter values and total number of blood meals, we get samples of the number of blood meals on each species from the multinomial process. With these samples of bird counts and blood meals, we calculated the feeding index for each sample, and thus produce a distribution of the feeding index.

Each host species’ population was divided into susceptible, infected, and recovered populations. The susceptible populations decrease due to infection. The infected populations increase due to the infection of susceptible hosts, and decrease due to recovery and natural death. Finally the recovered populations increase due to recovery of infectious hosts, and decrease due to natural death. The vector populations were split into susceptible and infectious populations. The susceptible vector populations also increase due to natural birth at rate, and decrease due to infection and natural death, while the infected vector population increases due to infection of susceptible hosts, and decrease due to natural death. Here it is assumed that the vector species do not recover from infection. Thus we have a system of 29 differential equations

$$\frac{\text{d}\text{S}_{\text{i}}}{\text{dt}}\text{=}{\text{-}\text{λ}}_{\text{b}_{\text{i}}}\text{S}_{\text{i}}\text{}$$

$$\frac{\text{d}\text{I}_{\text{i}}}{\text{dt}}\text{=}\text{λ}_{\text{b}_{\text{i}}}\text{S}_{\text{i}}\text{-}\text{γ}_{\text{b}}\text{I}_{\text{i}}\text{}$$

$$\frac{\text{d}\text{R}_{\text{i}}}{\text{dt}}\text{=}\text{γ}_{\text{b}}\text{I}_{\text{i}}\text{-d}\text{R}_{\text{i}}$$

$$\frac{\text{d}\text{I}_{\text{v}}}{\text{dt}}\text{=}\text{λ}_{\text{v}}\text{S}_{\text{v}}\text{-}\text{d}_{\text{v}}\text{I}_{\text{v}}\text{}$$

$$\frac{\text{d}\text{S}_{\text{v}}}{\text{dt}}\text{=}\text{N}_{\text{v}}\text{-d}\text{I}_{\text{v}}$$

with force of infection defined by

$$\text{λ}_{\text{b}_{\text{i}}}\text{=}\frac{v\text{β}_{1}\text{I}_{\text{v}}\text{α}_{\text{i}}}{\sum_{\text{j=1}}^{\text{n}} \text{α}_{\text{j}}\text{N}_{\text{j}}}\text{}$$

$$\text{λ}_{\text{v}}\text{=}\frac{\text{β}_{2}\sum_{\text{i=1}}^{\text{n}} \text{α}_{\text{i}}\text{I}_{\text{i}}}{\sum_{\text{j=1}}^{\text{n}} \text{α}_{\text{j}}\text{N}_{\text{j}}}$$

These equations were solved numerically using the LSODA routine from the ODEPACK library [1].

| Parameter, definition | Baseline value | Source |
| --- | --- | --- |
| d_v_: Vector mortality rate | .10 per day | imposed |
| β_1_: Vector- to host- transmission rate | 1 | 7 |
| β_2_: Host- to vector- transmission rate | Calculated | N/A |
| v: biting rate of vectors on host | .14 per day | 8,9 |
| γ_b_: Recovery rate of hosts | 1.0 per day | 7 |
| α_i_: Feeding index for host species i | Calculated | N/A |

**References:**

**1.** Hindmarsh, A. C. (2008). Brief Description of ODEPACK-A Systematized Collection of ODE Solvers Double Precision Version. *URL http://www.netlib.org/odepack/opkd-sum. Accessed*, *12*.

**2.** Morneau, F., Lépine, C., Décarie, R., Villard, M. & DesGranges, J. 1995 Reproduction of American robin (Turdus migratorius) in a suburban environment. Landscape Urban Plann. 32, 55–62. (doi:10.1016/0169-2046 (94)00177-5)

**3.** Farner, D. S. 1949 Age groups and longevity in the American robin: comments, further discussion and certain revisions. Wilson Bull. 61, 68–81.

**4.** Coulson, J. C. 1960 A study of the mortality of the starling based on ringing recoveries. J. Anim. Ecol. 29, 251–271. (doi:10.2307/2203)

**5.** Botkin, D. B. & Miller, R. S. 1974 Mortality rates and survival of birds. Am. Nat. 108, 181–192. (doi:10. 1086/282898)

**6.** Karr, J. R., Nichols, J. D., Klimkiewicz, M. K. & Brawn, J. D. 1990 Survival rates of birds of tropical and temperate forests: will the dogma survive. Am. Nat. 136, 277–291. (doi:10.1086/285098)

**7.** Komar N, Dohm DJ, Turell MJ, Spielman A. Eastern equine encephalitis virus in birds: relative competence of European starlings (*Sturnus vulgaris*). Am J Trop Med Hyg. 1999; 60: 387–391.

**8.** Wonham, M. J., de-Camino-Beck, T. & Lewis, M. A. 2004 An epidemiological model for West Nile virus: invasion analysis and control applications. Proc. R. Soc. Lond. B 271, 501–507. (doi:10.1098/rspb.2003.2608)

**9.** Vinogradova, E. B. 2000 Culex pipiens pipiens mosquitoes: taxonomy, distribution, ecology, physiology, genetics, applied importance and control, pp. 46–67. Soﬁa, Bulgaria: Pensoft.
